# Zebrafish as a Model to Unveil the Pro-Osteogenic Effects of Boron-Vitamin D3 Synergism

**DOI:** 10.3389/fnut.2022.868805

**Published:** 2022-04-29

**Authors:** Jerry Maria Sojan, Manu Kumar Gundappa, Alessio Carletti, Vasco Gaspar, Paulo Gavaia, Francesca Maradonna, Oliana Carnevali

**Affiliations:** ^1^Department of Life and Environmental Sciences, Università Politecnica delle Marche, Ancona, Italy; ^2^The Roslin Institute and Royal (Dick) School of Veterinary Studies, The University of Edinburgh, Midlothian, United Kingdom; ^3^Centro de Ciências do Mar (CCMAR), University of Algarve, Faro, Portugal; ^4^Faculty of Medicine and Biomedical Sciences, University of Algarve, Faro, Portugal

**Keywords:** bone, boron, vitamin D, micronutrients, zebrafish, RNA-Seq, transgenic lines, cell differentiation

## Abstract

The micronutrient boron (B) plays a key role during the ossification process as suggested by various *in vitro* and *in vivo* studies. To deepen our understanding of the molecular mechanism involved in the osteogenicity of B and its possible interaction with vitamin D3 (VD), wild-type AB zebrafish (*Danio rerio*) were used for morphometric analysis and transcriptomic analysis in addition to taking advantage of the availability of specific zebrafish osteoblast reporter lines. First, osteoactive concentrations of B, VD, and their combinations were established by morphometric analysis of the opercular bone in alizarin red-stained zebrafish larvae exposed to two selected concentrations of B (10 and 100 ng/ml), one concentration of VD (10 pg/ml), and their respective combinations. Bone formation, as measured by opercular bone growth, was significantly increased in the two combination treatments than VD alone. Subsequently, a transcriptomic approach was adopted to unveil the molecular key regulators involved in the synergy. Clustering of differentially expressed genes revealed enrichment toward bone and skeletal functions in the groups co-treated with B and VD. Downstream analysis confirmed mitogen-activated protein kinase as the most regulated pathway by the synergy groups in addition to transforming growth factor-β signaling, focal adhesion, and calcium signaling. The best-performing synergistic treatment, B at 10 ng/ml and VD at 10 pg/ml, was applied to two zebrafish transgenic lines, *Tg(sp7:mCherry)* and *Tg(bglap:EGFP)*, at multiple time points to further explore the results of the transcriptomic analysis. The synergistic treatment with B and VD induced enrichment of intermediate (*sp*7^+^) osteoblast at 6 and 9 days post fertilization (dpf) and of mature (*bglap*^+^) osteoblasts at 15 dpf. The results obtained validate the role of B in VD-dependent control over bone mineralization and can help to widen the spectrum of therapeutic approaches to alleviate pathological conditions caused by VD deficiency by using low concentrations of B as a nutritional additive.

## Introduction

Osteogenesis is a process that can be modulated by several factors, including macro- and micronutrient supplementation. Among the various micronutrients, it has been demonstrated that boron (B) has an important role in the development and maintenance of bone (1–3). B plays a crucial biological role in bone health by modulating the functions of various essential nutrients, including vitamin D3 (VD), calcium (Ca), and phosphorous (P), which are known to affect bone mineralization in both humans and animals (4–10). The presence of B is restricted to the mineral component of skeletal tissues and not to the organic matrix (11). Contrasting results were found in previous studies about the effect of B on the absorption of Ca and P, both essential nutrients for the skeletal formation. Dietary supplementation with B increased Ca and P absorption and balance in wethers and rats (12, 13) and promoted the improvement of the mechanical properties of bone tissue (14–18), while no such effects were observed in barrows (19). However, in vertebrates, B deficiency results in impaired osteogenesis, negatively affecting bone development (20, 21). Supplementation of B resulted in the improvement of bone strength and microstructure in mice (22). This was also observed in ostrich, where low concentrations of B supplemented through water led to increased osteogenesis through bone morphogenetic protein (BMP)-2 regulation (23). In addition, B was found to be able to reduce inflammation correlated with reduced bone mineral density, thereby improving the bone health (24), suggesting its use in the treatment of osteochondrosis (25). B supplementation was also able to enhance the fracture-healing process in rats (26). In addition to the *in vivo* evidence of the beneficial effects of B in the mineralization process, clear evidence was obtained *in vitro* as well. In pre-osteoblastic cell line MC3T3-E1, B was found to positively regulate mineralized tissue-associated proteins and messenger RNA (mRNA) expression of genes involved in osteoblastic functions such as osteocalcin (*bglap*), osteopontin (*spp1*), and collagen type 1 (27). In the same study, B concentrations of 10 and 100 ng/ml were able to increase *in vitro* mineralization in MC3T3-E1 cells (27). Similar concentrations were used in human bone marrow mesenchymal stem cells; although cell proliferation was not affected, an increased expression of BMPs and osteocalcin, along with elevation in the activity of alkaline phosphatase, was reported (28). Similarly, osteo-inductive properties of encapsulated B within scaffolds were confirmed in MC3T3-E1 cells (29).

Skeletal diseases such as osteoporosis, osteomalacia, and rickets are a significant medical burden worldwide, and VD deficiency is one of the major and perhaps the most preventable factor leading to bone fragility (30, 31). In a previous study, the supplementation of B in broiler chickens with VD deficiency alleviated symptoms associated with the insufficiency such as disruptions in the mineral metabolism (8). B supplementation further improved the biochemical characteristics such as Ca and P levels, thereby resulting in a healthier bone despite the VD deficiency (8). Similar observations were found in VD-deficient rats where B was able to increase Ca, Mg, and P (32). B interacts with VD probably by compensating the disturbances in the energy-substrate consumption or by enhancing the macro-mineral content of bone, in addition to the possibility of VD-independent regulation of the indices of maturation of cartilage (33). B was also shown to inhibit enzymes that catabolize VD, thereby causing an upregulatory impact on VD status (34). The exact mode of interaction between B and VD is not yet clear and needs further exploration.

Zebrafish (*Danio rerio*) is an increasingly relevant model for bone-related studies due to the many technical advantages associated with its use (35). In particular, translucent larval stages and cost-effective genetic manipulation translated into an increased availability of fluorescent reporter lines, which are particularly useful for *in vivo* cellular tracking, allowing the study of the fate and differentiation of specific bone cell types (36). Considering the necessity to provide valid solution to promote bone health, two concentrations of B were supplemented alone and in synergy with VD to zebrafish larvae and the impacts were studied at transcriptional level. Recent advances in next-generation sequencing guaranteed an easy access to RNA-sequencing data, which are being exploited here, and it allows the investigation of the action of compounds of interest on the bone metabolism and defines key pathways modulated by therapeutic interventions. To further validate the effects of B and VD on early skeletal development, *Tg(Ola.sp7:mCherry-Eco.NfsB)*^*pd*46^, and *Tg(Ola.bglap:EGFP)*^*hu*4008^, hereinafter mentioned as *Tg(sp7:mCherry)* and *Tg(bglap:EGFP)*, respectively. *D. rerio* lines were chosen for this study as sp7 is a zinc-finger-containing transcription factor expressed in pre-osteoblasts and immature osteoblasts, making it an excellent marker for cell tracking of osteoblastic cells (37), and bglap is a mature osteoblast marker, making it suitable to look into the effects of treatments on osteoblast maturation (36).

The analyzed data provide a greater understanding of the actions of these compounds in modulating the overall transcriptome with particular attention to the exact molecular regulation of skeletal development and time course of expression of sp7 and bglap proteins through transgenic lines. Furthermore, it opens the potential of using micronutrients like B as an additive to enhance the osteogenic efficiency of VD, thereby providing a low-cost solution to tackle the nutritional VD deficiency in biomedicine and aquaculture.

## Materials and Methods

### Wild-Type Zebrafish Husbandry, Experimental Design, and Alizarin Red S (AR-S) Staining

Adult wild-type AB female and male zebrafish specimens reared in the fish facility at Università Politecnica delle Marche (Ancona, Italy) were set up for overnight breeding at 2:1 ratio. The embryos were collected and divided into six groups in triplicates. Treatment concentrations were modified and adapted from previous studies (28, 38):

- Control: control group with ethanol at 0.1%.
- VD: VD group with VD3 (1α,25-dihydroxyvitamin D3; Sigma-Aldrich, USA) at 10 pg/ml.
- B10: B at 10 ng/ml.
- B10VD: B at 10 ng/ml with VD at 10 pg/ml.
- B100: B at 100 ng/ml.
- B100VD: B at 100 ng/ml with VD at 10 pg/ml.

Boric acid (Sigma-Aldrich, Germany) was used to make the concentrations of B across different treatment groups. The larvae were continuously treated *via* waterborne exposure with the respective compounds until 8 days post fertilization (dpf) and 70% of the water with respective treatments was renewed daily. VD was dissolved in ethanol before using in the treatments; therefore, ethanol (0.1%) was added to the control and two B groups without VD to ensure constant ethanol concentrations in all the groups. The sampling was done at 9 dpf for AR-S staining, image acquisition, and RNA extraction.

AR-S staining is one of the most used methods for studying bone mineralization (39). For fluorescence imaging, larvae (*n* = 5 per replicate per group) were exposed to an overdose of 300 mg/L MS-222 (ethyl 3-aminobenzoate methane sulfonate; Sigma-Aldrich, USA) and were stained with AR-S (Fluka Chemika, Switzerland) at 0.01% for 15 min. After washing with H_2_O, stained larvae were placed in a lateral position onto an agarose gel (2%). Images of the stained larvae were taken using a Zeiss Axio Imager M2 fluorescent microscope (Milan, Italy) set with a green light filter (λex = 530–560 nm and λem = 580 nm). Images were acquired using constant parameters and analyzed using ImageJ (version 2.1.0/1.53c) software after splitting the color channels of the RGB images. Eight-bit images were adjusted uniformly for all the images to achieve optimum contrast and brightness for improved visibility of the operculum bone.

### Zebrafish Transgenic Lines Husbandry, Experimental Design, and Image Analysis

Broodstock from the transgenic lines used in our experiments, *Tg(sp7:mCherry)* and *Tg(bglap:EGFP)*, were maintained in a recirculating water system (Tecniplast, Italy) at the aquatic animal experimental facilities of the Centre of Marine Sciences, Faro, Portugal. Eggs were produced with an in-house breeding program and maintained in static conditions until hatching at 3 dpf. Larvae were then screened with a Leica MZ10F fluorescence stereomicroscope (Leica, Germany) and 400 fish expressing the reporter proteins were selected and randomly distributed into four 300 ml beaker cups (100 fish/beaker) with the respective treatments in water. The selected four experimental groups were

- Control: control group with ethanol at 0.1%.
- VD: VD group with VD3 (1α,25-dihydroxyvitamin D3, Sigma-Aldrich, USA) at 10 pg/ml.
- B10: B at 10 ng/ml.
- B10VD: B at 10 ng/ml with VD3 at 10 pg/ml.

Boric acid (USB Corporation, USA) was used to make the required concentrations of B groups. Fish (*n* = 20) were sampled at four different time points, 6, 9, 12, and 15 dpf, stained with 0.01% AR-S (Sigma-Aldrich, USA) or 0.1% calcein (Sigma-Aldrich, USA) to label mineralized structures and imaged using a Leica MZ10F fluorescence stereomicroscope equipped with a green fluorescence filter (λ_ex_ = 546/10 nm) and a barrier filter (λ_em_ = 590 nm) for *Tg(sp7:mCherry)* and AR-S stained fish; and with a blue fluorescence filter (λ_ex_ = 470/40 nm) and a barrier filter (λ_em_ = 515 nm) for *Tg(bglap:EGFP)* and calcein-stained fish. All images were acquired with a DFC7000T color camera (Leica, Germany) according to the following parameters, namely, 24-bit colored image, exposure time 2 s (green channel) and 1 s (red channel), gamma 1.00, image format 1920 × 1440 pixels, and binning 1 × 1. Fluorescence images were processed with ZFBONE macro toolset for Fiji (40).

### RNA Extraction and Quantification

At 9 dpf, the larvae were sampled using an overdose of MS-222 and stored at −80°C. There were three biological replicates for each experimental group, and each replicate consisted of a pool of seven larvae. Total RNA was extracted from each replicate sample using RNAeasy® Minikit (QIAGEN, Germany) and eluted in 20 μl of molecular grade nuclease-free water. Final RNA concentrations were determined using a nanophotometer (Implen, Germany). Total RNA was treated with DNase (10 IU at 37°C for 10 min; Sigma-Aldrich, USA), and quality was confirmed using gel electrophoresis (1% gel) and stored at −80°C until library preparation for RNA sequencing. iScript cDNA Synthesis Kit (Bio-Rad, USA) was used to perform cDNA synthesis using 1 μg of total RNA and stored at −20°C until further use in real-time PCRs (RT-PCR).

### RNA Sequencing and Quality Control

Samples to be used for RNA sequencing were confirmed for concentration using Invitrogen Qubit 3.0 Fluorometer with the RNA assay kit and for integrity using Agilent Tapestation. Illumina TruSeq RNA libraries were prepared by Novogene Ltd. (Cambridge, UK) and sequenced on an Illumina Novaseq6000. All the triplicate samples of each group were sequenced to generate approximately 30 million paired end reads of 150 base pairs (bp) each (Supplementary Table 1). The read data were assessed for its quality using FastQC version 0.11.5 (http://www.bioinformatics.babraham.ac.uk/projects/fastqc/). Reads were then trimmed using TrimGalore version 0.4.4 (https://github.com/FelixKrueger/TrimGalore) by setting the parameters -q 30, -stringency 5, -length 40. Specifically, reads were trimmed for any adapters, and bases with a Phred score of <25 were trimmed off. Trimmed reads <40 bp were also removed.

### Differential Expression Analysis

The final cleaned up reads were then mapped to the *D. rerio* reference genome (GRCz11) retrieved from Ensembl genome database. Mapping was performed using STAR aligner (41) with the parameters (–outSAMtype BAM SortedByCoordinate, –outSAMunmappedWithin, –outSAMattributes Standard). Gene-level read count data was generated using featureCounts (42) with the parameters (–primary, -C, -t exon, -g gene_id), and the rest were set to default.

Differential gene expression was performed using DESeq2 1.26.0 (43) within R 3.6.1 (44). Finally, genes with false discovery rate < 0.05 and absolute log2 fold change values (FC) >0.5 were considered as differentially expressed. Principal component analysis (PCA) plots were generated to remove any outlier samples from the data using plotPCA function within DESeq2 1.26.0 followed by hierarchical clustering across samples using the function heatmap.2 within the package gplots 3.0.1.1 (45) to confirm the clustering of replicates. A list of differentially expressed genes (DEGs) was generated for all treatment combinations and concatenated to generate a final list of genes that were differentially expressed in at least one combination. The DEGs were then clustered using partition around medoids (PAM) algorithm (46) into different clusters based on the DESeq2 median ratio normalized expression values across different treatments using the package cluster 2.1.0 (47). The optimum number of clusters was identified using Gap statistic method (48) within the package factoextra 1.0.6 (49). The normalized counts were mean centralized across different treatments within each cluster and visualized using the package ggplot2 3.2.1(50).

Gene set enrichment analysis was performed using Clusterprofiler 3.14.3 package (51). Annotations for *D. rerio* were retrieved from the package org.Dr.eg.db 3.8.2 (52), and gene set enrichment analysis was performed for DEGs in the clusters individually. Gene Ontology (GO) terms falling under the categories Biological Process (BP), Cellular Component (CC), and Molecular Function (MF) with a *p* < 0.05 were considered as significant and used for downstream analysis. Enriched GO terms and genes within each cluster were then used to generate bipartite networks using the package ggnetwork 0.5.1 (53) within R. Kyoto Encyclopedia of Genes and Genomes (KEGG) pathway enrichment analysis was performed against the DEGs present in the three clusters and filtered for *p* < 0.05 to be considered significant. Bubble plots for enriched KEGG pathways across all the clusters were generated using ggplot2 3.2.1.

### RT-PCR

RT-PCRs were performed with SYBR green in a CFX thermal cycler (Bio-Rad, Italy) in triplicate as previously described (54). The thermal profile for all reactions was 3 min at 95°C followed by 45 cycles of 20 s at 95°C, 20 s at 60°C, and 20 s at 72°C. Dissociation curve analysis showed a single peak in all the cases. Ribosomal protein L13a (*rpl13a*) and ribosomal protein, large, P0 (*rplp0*) were used as the housekeeping genes to standardize the results by eliminating variation in mRNA and cDNA quantity and quality. No amplification product was observed in negative controls and primer-dimer formation was never seen. Data were analyzed using iQ5 Optical System version 2.1 (Bio-Rad), including Genex Macro iQ5 Conversion and Genex Macro iQ5 files. Modification of gene expression between the groups is reported as relative mRNA abundance (arbitrary units). Primers were used at a final concentration of 10 pmol/ml. Primer sequences are listed in Supplementary Table 2.

### Statistical Analysis

Data of all groups were normally distributed as assessed using Shapiro-Wilk's test (*p* > 0.05), and there was homogeneity of variances, as assessed using Levene's test for equality of variances (*p* > 0.05). The differences between the control and the treatments were tested with a one-way analysis of variance (ANOVA) followed by Tukey's *post-hoc* test (*p* < 0.05) for the experiments with the transgenic lines and the image data of AR-S staining. All the tests were performed using R version 3.6.1(44), and plots were generated using ggplot2 3.2.1.

## Results

### Increased Operculum Bone Calcification in Synergy Treatment Groups

**Figure 1:**
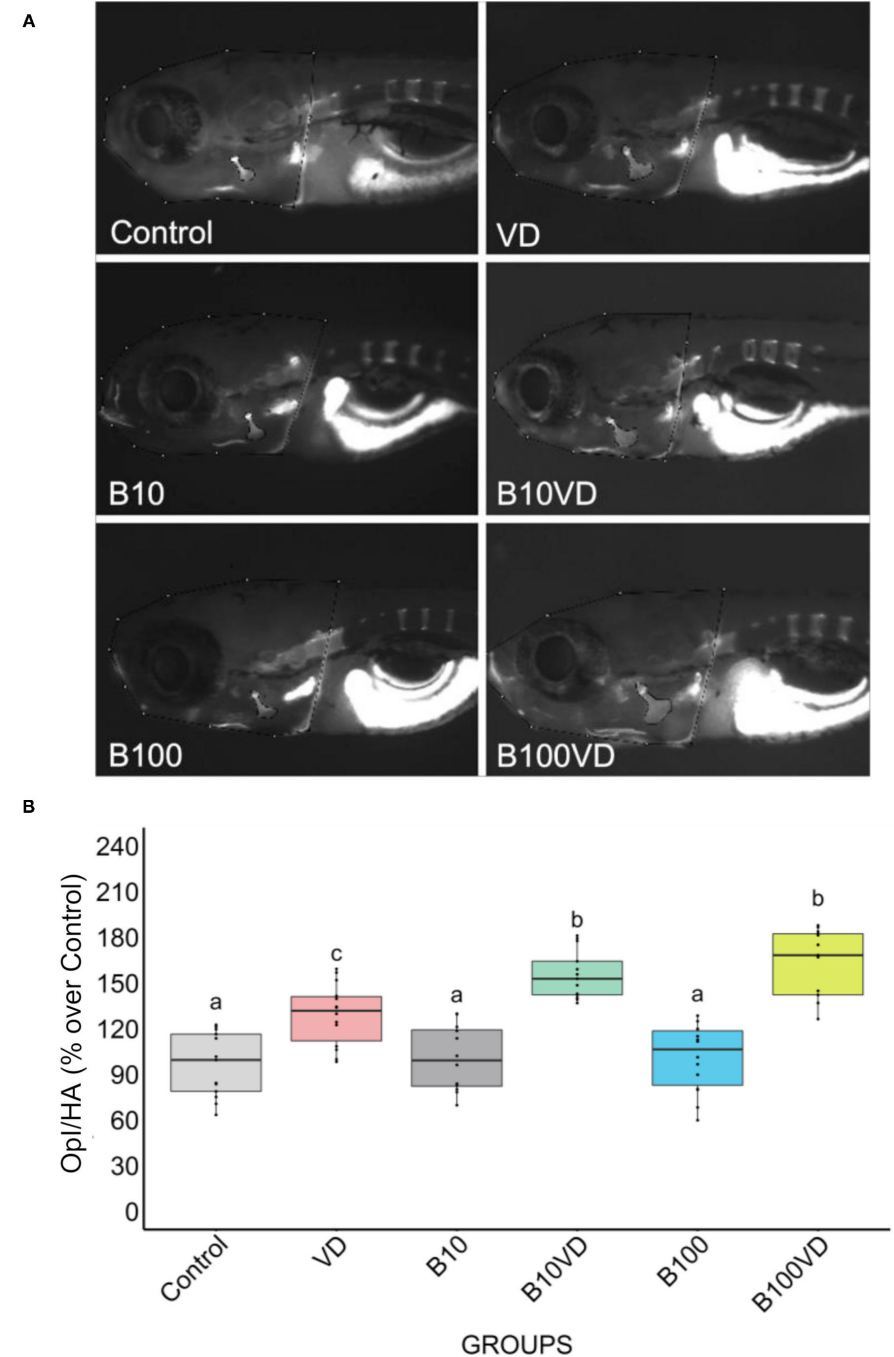
Fluorescence microphotograph of AR-S-stained larvae and ImageJ quantification of the opercular bone mineralization. **(A)** Mineralized bones stained by AR-S staining at 9 dpf following different treatments. **(B)** Quantitative analysis of the operculum bone integrated pixel density normalized by head area (Opl/HA) showed as % over control in fish treated with different concentrations of B and VD. Different letters above each graph indicate statistically significant differences among different groups. One-way ANOVA and Tukey's *post-hoc* test were used, and statistical significance was set at *p* < 0.05.

No major differences in opercular bone growth were observed between the two concentrations of B (B10 and B100), whereas an increase in mineralization of the opercula was observed in groups treated with VD with respect to control, B10, and B100. However, both the synergy groups (B10VD and B100VD) showed an increase in mineralized area of the opercular bone when compared to VD treatment (Figure 1A). Quantitative analysis of the integrated pixel density within the operculum area was performed as it adequately proxies the intensity of fluorescence signal, thus providing an index of the density of AR-S staining (i.e., mineralization). Using ImageJ (version 2.1.0/1.53c), the area of the operculum (OpA) and the area of the head (HA) were manually selected and the raw integrated density within the area of the operculum (Opl) was extracted. Opl was then normalized with the area of the head to compensate for differences in size among fish. Normalized pixel density within the operculum (Opl/HA) did not vary among fish treated with ethanol (control) and the two concentrations of B (B10, B100) while VD and both synergy groups significantly increased mineralization of the operculum with respect to the control group and both B concentrations (Figure 1B). Importantly, opercular bone mineralization was significantly increased in fish treated with both synergy groups (B10VD, B100VD) compared to VD in what appeared to be a dose-dependent manner, although no significant differences were found between the synergy groups with different concentrations of B (Figure 1B).

### Differential Expression Analysis and Clustering of DEGs

**Figure 2:**
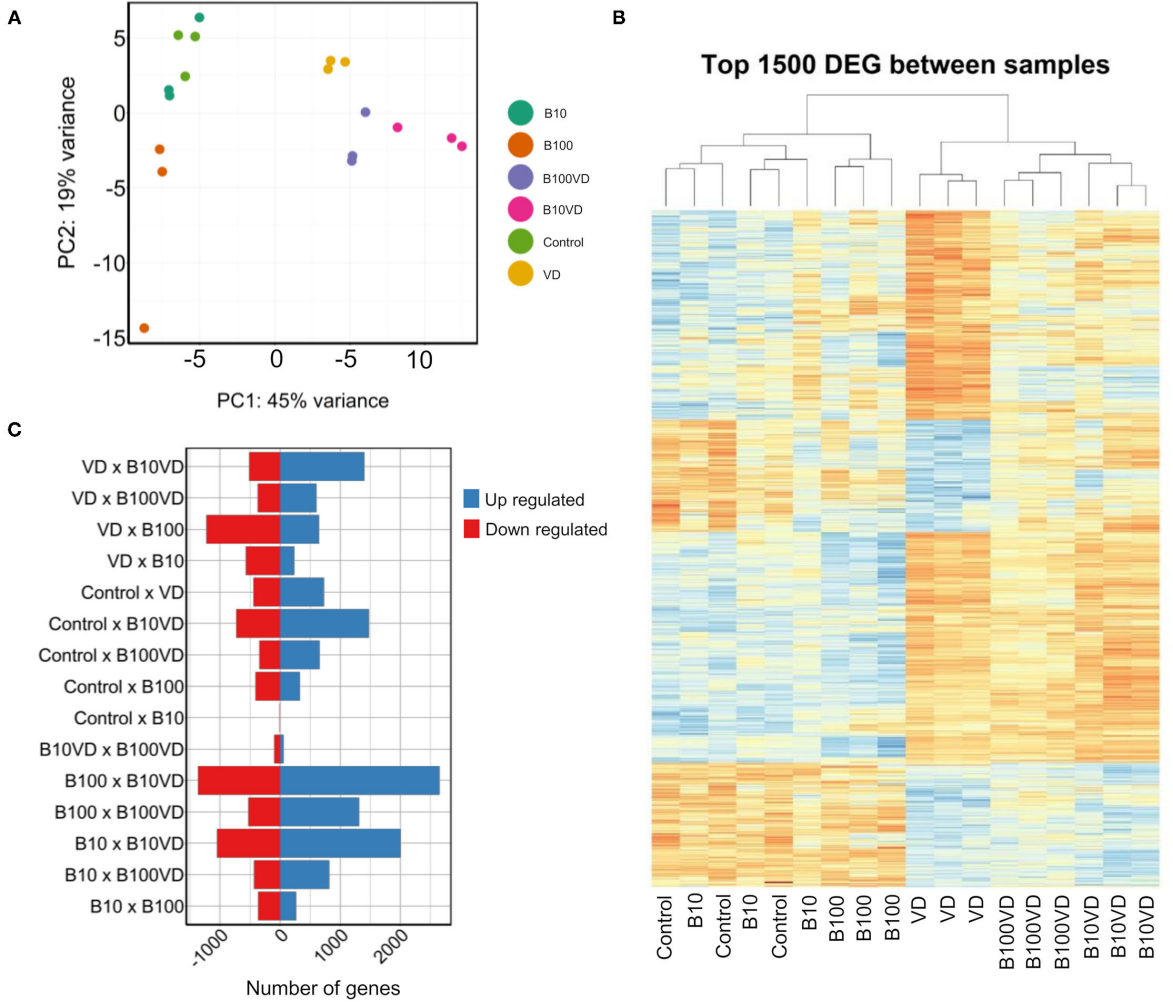
Sample clustering and DEGs across different combinations. **(A)** PCA of all the samples used in the experiment. **(B)** Hierarchical clustering of the top 1,500 DEGs across all the samples used in the experiment. **(C)** Number of upregulated and downregulated genes across different combinations of experimental groups.

Approximately 30 million paired end reads of 150 bp were generated across each sample of RNA in triplicates from each treatment group at 9 dpf. PCA on the read count data stratified the different treatments and replicates into distinct clusters (Figure 2A). Hierarchical clustering of the top 1500 DEGs clustered the different replicates of different treatments together (Figure 2B), confirming uniformity among the replicates. However, control and B10 samples showed an interspersing in both the PCA and hierarchical clustering, indicating very low variability in expression between these groups, which is also consistent with the dimensions of the operculum between the two treatment groups (Figure 1B). Differential expression analysis on all these groups combined resulted in a set of 7,341 genes differentially expressed in at least one of the different contrasts listed in Figure 2C and Supplementary Table 3. Comparing the differential expression of different treatments against control revealed B10VD to be a highly responsive group against control with 1,477 and 724 genes up- and downregulated, respectively (Figure 2C). B10 treatment in contrast just revealed four downregulated and one upregulated gene, showing to be the treatment with the lowest transcriptional response against control (Figure 2C).

**Figure 3:**
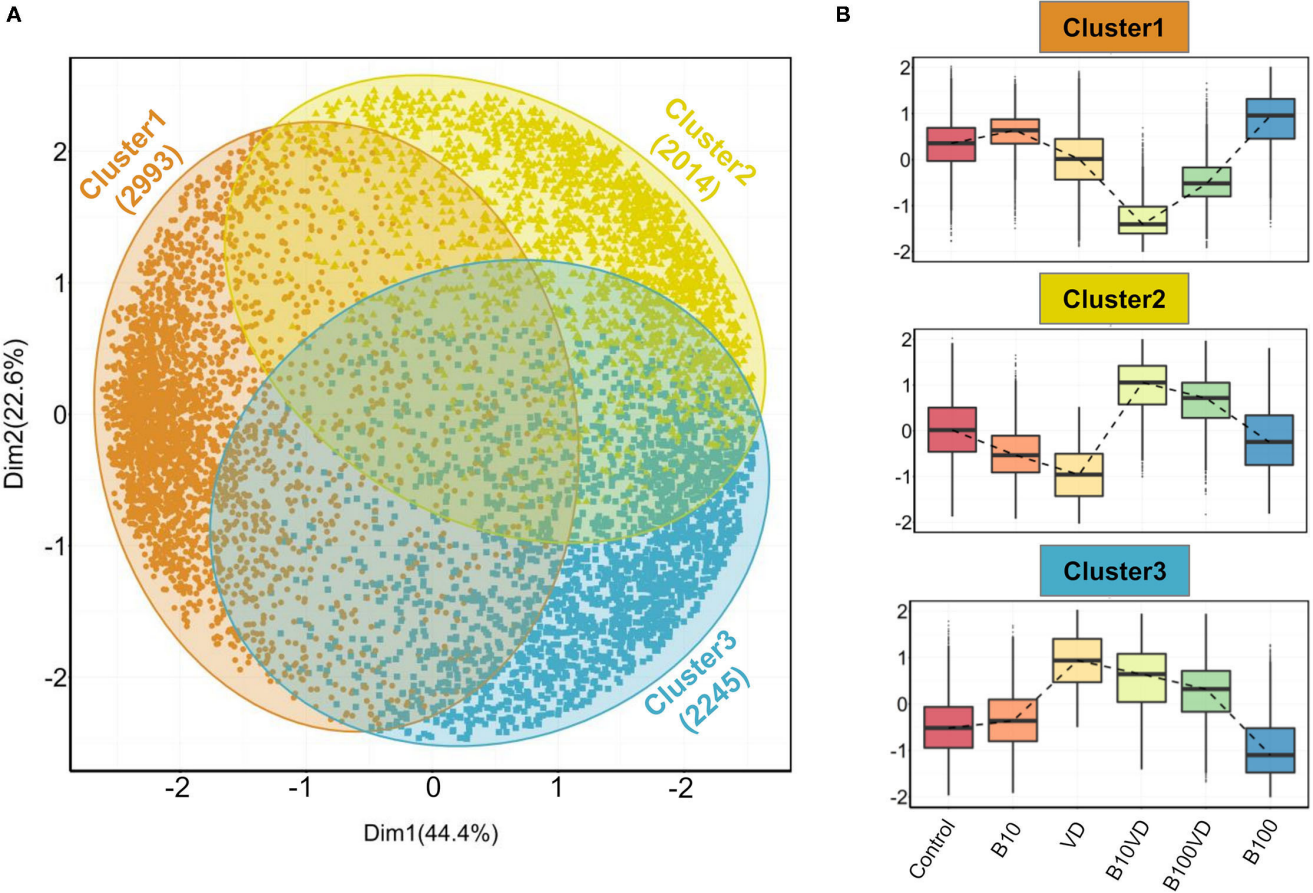
Outputs from PAM clustering of all the DEGs. **(A)** PCA visualizing the three clusters labeled cluster 1, cluster 2, and cluster 3 (indicated in orange, yellow, and blue colors, respectively) as defined by the PAM clustering. **(B)** Expression patterns of normalized gene expression values across different treatment groups in each of the three clusters. The expression counts were mean-centered before plotting them as box plots. The dashed lines within each panel connect the median values across different treatment groups.

Gap statistic method determined three clusters to be optimum for PAM clustering (Supplementary Figure 1). PAM clustering defined three clusters C1, C2, C3 with 2,993, 2,014, and 2,245 genes in each cluster (Figure 3A), respectively. The three clusters revealed distinct expression patterns across different treatments, and normalized expression values in cluster 1 were highest for B100 and lowest for B10VD (Figure 3B). Cluster 2 genes displayed a contrasting expression pattern between VD (decreased) and B10VD (increased) compared to control, while cluster 3 harbored genes showed increased expression in VD, B10VD, B100VD groups compared to the control and other treatment groups.

### Functional Annotation of DEGs

**Figure 4:**
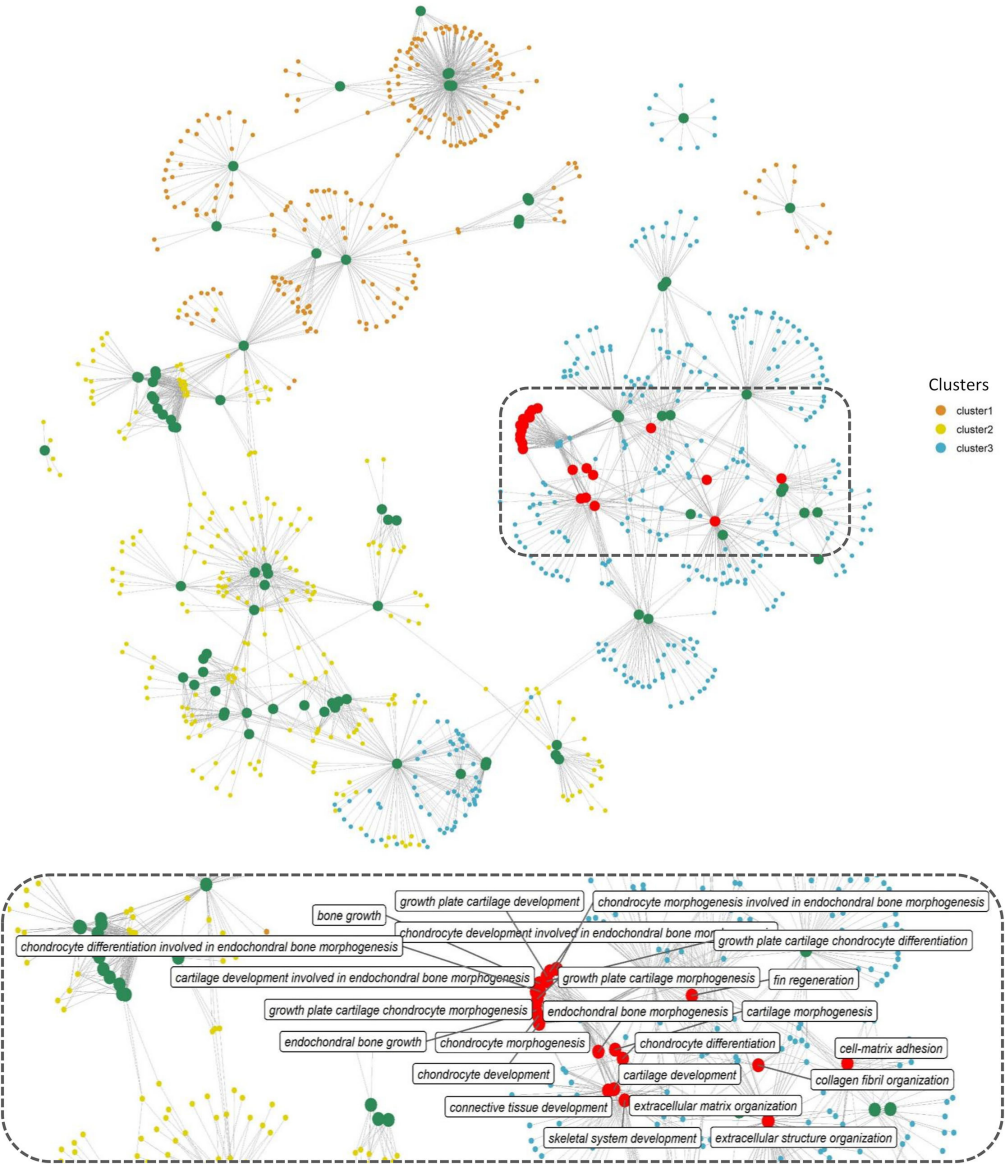
Network representation of enriched GO terms (BP) performed against the DEGs across three different clusters (Supplementary Table 4), the large-sized green and red-colored nodes indicate enriched GO terms, the red nodes in particular highlight GO terms in bone and skeleton system functioning, the region within the dashed rectangle is zoomed at the bottom of the network to highlight the nomenclature of key GO terms. Each small node of orange, green, and blue colors indicates a gene contributing to the enriched GO term across different clusters.

Gene function enrichment analysis for all the DEGs across each cluster revealed distinct functional enrichment for GO terms and KEGG analysis. Cluster 1 was enriched for a total of 47 GO terms across BP, MF, and CC categories with a very high representation of terms involved in catabolic processes (GO:0019941, GO:0010499, GO:0043632, GO:0006511) and proteasome assembly (GO:0043248) (details of GO terms are given in Supplementary Table 4). A total of 117 GO terms were enriched in cluster 2, and there was no overrepresentation of GO terms contributing to a particular function across all the three GO categories (Supplementary Table 4). Cluster 1 and cluster 2 revealed no significant enrichment for functions or processes related to skeletal system. Interestingly, cluster 3 stood out among the three with a high enrichment for GO terms involved in bone and skeleton system functioning (Supplementary Table 4). In cluster 3, a total of 44 GO terms were enriched in the BP category out of which 23 GO terms were involved in processes, leading to bone and skeletal system functioning, such as extracellular matrix (ECM) organization (GO:0030198; *p* = 1.29e−16) and skeletal system development (GO:0001501; *p* = 0.000504). In addition, within cluster 3, GO terms involved in skeletogenesis were again enriched in the CC (<10 terms) and MF (<5 terms) category (Supplementary Table 4); however, the key focus was diverted toward BP considering more than half of the enriched processes were related to bone and skeletal system. The bipartite network showing interactions of genes and enriched GO terms (BP) across the clusters displayed minimum overlap between enriched GO terms across three different clusters (Figure 4). This further indicates that genes showing distinct expression profiles across the three clusters are in fact enriched for distinct functions. Also, many genes in cluster 3 are shared across multiple GO terms with bone and skeletal system functions as highlighted in Figure 4, which otherwise is not observed in the other two clusters.

**Figure 5:**
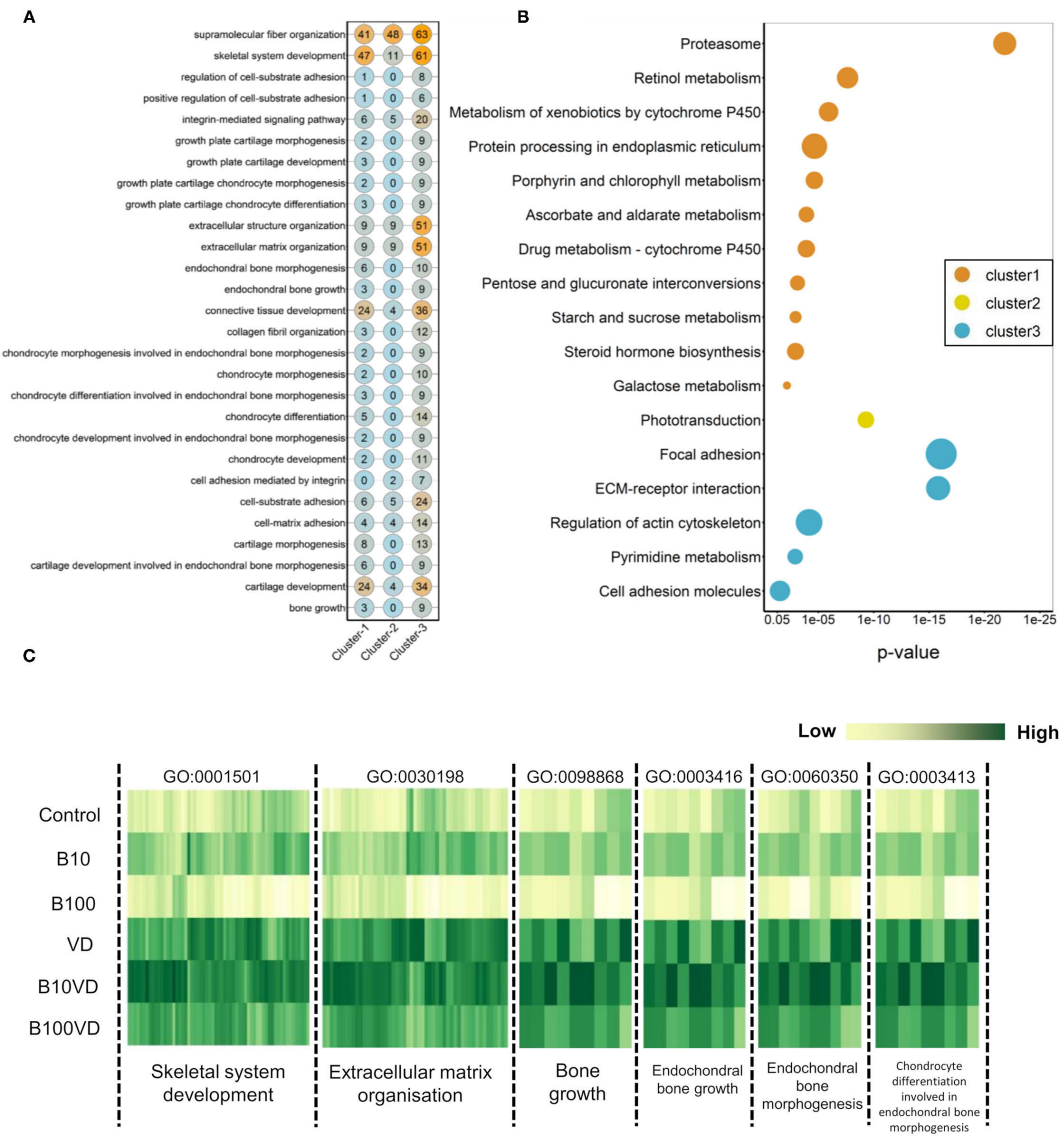
**(A)** Matrix visualizing the number of DEGs falling under enriched GO terms involved in skeletal system development. Y-axis highlights the key enriched GO terms, and X-axis describes the cluster number. The numbers within each circle indicate the total genes falling within that GO term in each cluster. **(B)** Bubble plot visualizing KEGG pathways significantly enriched (*p* < 0.05) across the three different clusters. Color of the bubbles indicates the cluster they fall into, and size of the bubble indicates the number of genes. **(C)** Heatmap visualizing normalized expression values of genes contributing to enriched skeletal GO terms in the cluster 3. Y-axis highlights the different treatment groups, and X-axis describes the enriched GO terms.

We further investigated the strong enrichment for skeletal GO terms (BP) by generating a quantitative matrix of the number of genes falling within each GO term across three clusters (Figure 5A). Increased number of genes involved in skeletal functions within cluster 3 correlates well with the strong enrichment in GO terms. KEGG analysis revealed cluster 1 to be enriched for 11 KEGG pathways, while cluster 2 displayed enrichment for only one pathway, phototransduction, involving 21 genes (Figure 5B; Supplementary Table 5). Cluster 3, which showed a strong bias toward skeletal functions in GO analysis, was enriched for five KEGG pathways, out of which focal adhesion (dre04510), ECM-receptor interaction (dre04512), and regulation of actin cytoskeleton (dre04810) contribute toward bone/skeletal functions (Figure 5B). Heatmaps showing normalized expression values of genes falling within the key enriched bone and skeletal-related GO terms such as skeletal system development (GO:0001501), ECM organization (GO:0030198), bone growth (GO:0098868), endochondral bone growth (GO:0003416), endochondral bone morphogenesis (GO:0060350), and chondrocyte differentiation involved in endochondral bone morphogenesis (GO:0003413) revealed an increased expression in the B10VD group compared to all other groups (Figure 5C). There is a bias toward increased expression of skeletal system genes in B10VD despite the overall expression pattern of cluster 3 showing the highest expression for the VD group (Figures 3A, 5C).

A total of 354 genes related to bone and skeletal system were identified from the 28 GO terms listed in Figure 5A. In comparison to control, 101 DEGs for VD, 126 DEGs for B10VD, and 64 DEGs for B100VD were found. Since we observed more DEGs with B10VD, which is the synergy group with less concentration of B, downstream analysis was focused on B10VD alone. A total of 55 genes (3 downregulated and 52 upregulated) were commonly differentially expressed for B10VD and VD compared to control. In addition, 71 DEGs were unique for B10VD and 46 were specific for VD. Some important genes like *col1a1a* and *ucmab* were common DEGs among B10VD and VD, whereas *dcn* was upregulated only in B10VD. The osteoclast marker gene *ctsk* (cathepsin K), on the contrary, was found to be upregulated only in VD and not in B10VD.

We further decided to investigate synergy by exploring the KEGG pathways that were not enriched in the current analysis but were key for bone and skeletal development in zebrafish. KEGG pathway maps for mitogen-activated protein kinase (MAPK) (dre04010), transforming growth factor beta (TGF-β) (dre04350), focal adhesion (dre0510), WNT signaling pathway (dre04310), and Ca signaling (dre04020) were generated incorporating the log FC data for B10VD, VD treatments in contrast to control (Supplementary Figure 2). Expression patterns of the genes from these pathways further confirmed B10VD synergy to be more effective in supporting bone and skeletal development. Key candidate genes from these pathways such as *cacn3b, egfra, mapk14b, mras, ppp3cca*, and *rps6ka3b* were confirmed for their upregulation in B10VD than VD or B100VD (Supplementary Table 3; Supplementary Figure 2).

### RT-PCR Validation of RNA-Seq Data Using Selected Genes From MAPK Pathway

Given their importance for osteogenesis and mineralogenesis, eight marker genes (*cacn3b, dusp2, egfra, hspb1, mapk14b, mrasb, ppp3cca, rps6ka3b*) involved in the MAPK pathway were selected for validation of the transcriptomic data by RT-PCR. As shown in Supplementary Figure 3, the relative FC in RT-PCR was consistent with RNA-Seq results, suggesting that the transcript identification and quantification were extremely consistent between the two techniques. Most genes from RNA-Seq analysis were in good accordance with the expression intensities by RT-PCR, although the result was dissimilar for *mapk14b* in the VD group, probably due to the difference in sensitivity of each technique.

### Time-Course Study of Operculum Bone Growth Using the Transgenic Lines *Tg(sp7: mCherry)* and *Tg(bglap:EGFP)*

**Figure 6:**
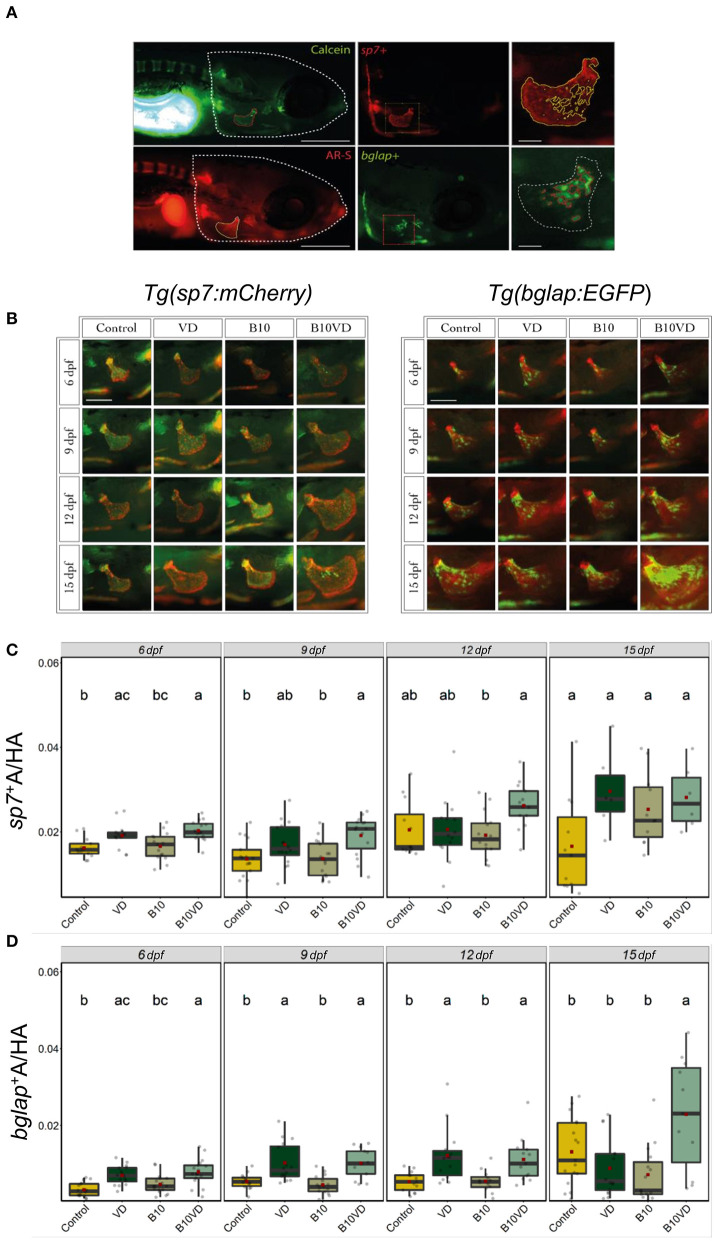
Early and mature osteoblasts respectively tracked by the fluorescence expression of *Tg(sp7:mCherry)* and *Tg(bglap:EGFP)*. Four groups of treatment are ethanol at 0.1% (Control), VD group with VD3 at 10 pg/ml (VD), B at 10 ng/ml (B10), B at 10 ng/ml with VD3 at 10 pg/ml (B10VD). **(A)** An example of how the areas of the operculum and *sp*7^+^ and *bglap*^+^ were measured (big scale bar = 0.35 mm; small bar = 0.05 mm). **(B)** A table with merged pictures of operculum from the sp7:mCherry line stained with calcein and the *bglap*:EGFP line stained with AR-S (scale bar = 0.17 mm). **(C)** The *sp*7^+^ area inside the operculum was normalized with the total head area at time points 6, 9, 12, and 15 dpf. **(D)** The *bglap*^+^ area inside the operculum was normalized with the total head area at time points 6, 9, 12, and 15 dpf. One-way ANOVA and Tukey's *post-hoc* test were used. Data are presented as means ± SD, and different letters represent statistical significance at *p* < 0.05.

Since B10VD is the synergy group with a lower concentration of B, yet with more DEGs, to further investigate the synergy effect at various stages of skeletal development, additional analysis was performed at multiple time points using two zebrafish fluorescent reporter lines, one expressing mCherry under the control of the medaka (*Oryzias latipes*) *sp7* (osterix) promoter, the other expressing EGFP downstream to the promoter of medaka *bglap* (osteocalcin) (Figures 6A,B). Areas of the operculum and *sp*7^+^ and *bglap*^+^ areas, showing early and mature osteoblasts, respectively, were measured (Figures 6C,D). Synergy group (B10VD) exhibits the largest mineralized area of the operculum as well as the largest *sp*7^+^ and *bglap*^+^ areas at all the time points analyzed (Figures 6C,D). The *sp*7^+^ area normalized with the head area (*sp*7^+^A/HA) was significantly higher in VD and B10VD with respect to control at 6 dpf, whereas at 9 dpf only the synergy group showed a significantly higher fluorescence signal with respect to control. At 12 and 15 dpf, B10VD remained the group with the largest *sp*7^+^ area, but significant differences were only found with the B group at 12 dpf. No differences between the control and B groups were detected throughout the experiment (Figure 6C). GFP fluorescence marking mature osteoblasts normalized with head area (*bglap*^+^A/HA) showed a significant increase for the synergy group compared to every other group at 15 dpf. Importantly, this time point was characterized by the strongest signal among all end points studied. Synergy group (B10VD) also showed a significantly larger *bglap*^+^ area than control at every time point studied, whereas VD showed a larger *bglap*^+^ area than control at all time points except 15 dpf. Synergy group also showed a significantly stronger signal compared to VD or B alone at 15 dpf. In accordance with what was observed for *sp7* expression pattern, no differences were detected between control and B at any of the end points evaluated. Except VD, all other groups showed a general increase of *bglap*^+^ area from 6 to 15 dpf (Figure 6C).

## Discussion

Morphometric assessment of the mineralizing opercular bone in early-stage zebrafish larvae was previously described as a suitable tool for the screening of osteogenic compounds (38, 55). The pro-osteogenic properties of VD are known since a long time but the possible synergistic actions of VD with micronutrients to promote bone mineralization are understood less. Here we tested two concentrations of B, one concentration of VD and their respective combinations, and observed the induction of osteogenic effect as observed by increased mineralized operculum areas in both the synergy groups compared to the supplementation of VD alone but even more pronounced effect when compared to the control and the B treatments alone. Following this preliminary screening, and with the scope of exploring the molecular determinants of the observed phenotype, we analyzed the transcriptome of the treated larvae to obtain a large multivariate dataset of DEGs with particular attention given to pathways associated with the skeletal system. The PAM clustering approach was previously proven to be appropriate to cluster and define expression patterns among the DEGs (56) and the same was observed here, where a dominance of skeletal- or bone-related pathways was observed in one out of the three clusters. This cluster of interest (C3) had a specific pattern for groups with VD, where VD and its synergy groups stayed in the high-expression side, whereas control and groups with B stayed in the low-expression side. Interestingly, when looking at the expression patterns of genes contributing to enriched skeletal function GO terms, only B10VD showed higher expression than the VD group, suggesting a potential role that the synergy between VD and B could be playing in inducing the pro-mineralogenic effect observed. In addition, transcriptomic responses showed that more genes with positive effects on the skeleton were highly expressed in the combination group of VD with a lower concentration of B (B10VD) than the other synergy group with a higher concentration of B (B100VD). Thus, on a molecular level, 10 ng/ml B with VD showed the strongest positive effect on metabolic pathways associated with skeletal development.

Among B10VD and VD, there were common DEGs, including *col1a1a*, expressed in developing bony elements and ectoderm, and *ucmab*, which play a pivotal role in zebrafish skeletal development (57, 58). Some important genes were expressed only for B10VD such as *dcn*, which encodes for an important ECM glycoprotein that has a role in regulation of bone mass by modulating TGF-β activity (59). The most affected pathway was observed to be the MAPK signaling, where many genes were found to be overexpressed in the synergy groups compared to VD. RSK2 (encoded by *rps6ka3a* gene) was shown to be highly upregulated in the B10VD group relative to B100VD and VD groups, which is particularly relevant, given its importance for osteoblast differentiation and function (60). It is in fact involved at the distal end of the MAPK pathway, where it plays a key role in bone turnover by phosphorylating different substrates, such as cAMP response element-binding protein, which is a known inducer of osteoblast differentiation and c-Fos, which is an osteoclast differentiation inducer (61–63). C-Fos was found to be downregulated in both VD and B10VD, but osteoclast marker gene *ctsk* was upregulated only in VD. This indicates that the synergy group possibly has a reduced VD-induced osteoclast formation through VD receptors in the nuclei as found in previous studies (64). The synergy groups also showed significant upregulation for p38, which is involved in the *runx2* activation (65), giving further evidence to the pro-osteogenic effect of B and VD synergy in the induction of osteoblast differentiation. *Fgfr4* receptor, a gene in the ERK signaling pathway that is involved in the *runx2* transcriptional activation, was also found to be highly upregulated in the synergy groups, pointing out at a possibility of *runx2* regulation, and thereby to be involved in the differentiation and proliferation of osteoblasts (66, 67). Although *runx2* was not one of the DEGs observed here and that could be due to the specific skeletal development stage of the larvae under study. *Fgfr4*, being the receptor for *fgf6*, also plays a pivotal role in osteoblast and osteoclast differentiation (68). The calcineurin, which is part of multiple bone-related pathways such as MAPK, WNT, and Ca signaling, was found to be upregulated in the B10VD synergy group. Calcineurin is known to be expressed in osteoblasts and enhances the differentiation of osteoblasts, thereby increasing bone formation (69). The focal adhesion pathway was also regulated in both the synergy groups where fibronectin 1 (*fn1*) was highly upregulated. *fn1*, through activation of the WNT pathway, stimulates osteoblast differentiation and ECM mineralization as observed in previous *in vitro* studies, and it is known to be produced by osteoblasts during bone generation (70, 71). Additionally, *fn1* was found to play a role in osteoblast compaction through fibronectin fibrillogenesis cell-mediated matrix assembly, which is crucial for the bone ECM mineralization mediated by osteoblast (72, 73). In the KEGG analysis of the TGF-β/BMP pathway, *smad4* was largely upregulated in the B10VD group. Proteins belonging to the Smad family are associated with the BMP pathway, whose activation is paramount for bone mineralization and osteoblast differentiation (74, 75). The transcriptomic analysis clearly evidenced the upregulation of genes involved in bone formation by osteoblast differentiation, ECM formation, and mineralization in the synergy groups, particularly in B10VD, with a higher effect than VD treated alone.

From the combined morphological and transcriptomic data, we decided to further investigate the synergy of VD with B at a lower concentration, 10 ng/ml. To explore the effect of the B-VD synergy on bone development in a time-dependent manner, cellular dynamics of intermediate and late osteoblasts, as labeled by *sp7* and *bglap* expression, respectively, were verified. Sp7 is a highly conserved, zinc finger-containing transcription factor essential within the stepwise genetic program regulating osteoblast differentiation. It is required for the activation of a repertoire of genes directly associated to osteoblast maturity and bone ECM formation and mineralization such as *bglap, spp1, col10a1a/b*, and *sparc* (76, 77). In zebrafish, *sp7* expression is considered to be labeling intermediate stages of osteoblast differentiation and tends to be downregulated in mature osteoblasts (76). It is first observed at the time of the onset of the primary cranial ossified structures (36 hpf), with its expression pattern perfectly co-localizes with the whole mineralized domain of the bone (76). In contrast, Osteocalcin (Bglap) is a small osteoblast-secreted protein well accepted as a marker of mature and matrix-secreting osteoblasts (78). Plenty of evidence suggests that VD exerts a direct stimulating effect on osteoblasts proliferation and differentiation through the VD receptor in humans and other mammals (79). Similarly, it has been previously observed in zebrafish that treatment with VD is able to increase the expression of *sp7* and *blglap* at very early stages of skeletal development (6 dpf) (38). Accordingly, in this study a significant increase in the area of *sp*7^+^ cells within the opercular bone was observed for VD alone and when in combination with B at early stages of opercular development (6 dpf), but only the synergy group increased the amount of intermediate osteoblast (*sp*7^+^) populating the operculum at 9 dpf. Similarly, when looking at more advanced (*bglap*^+^) osteoblasts stages, both VD alone and synergy group increased *bglap*^+^ osteoblasts in early stages (6, 9, and 12 dpf), but the synergy group extended the induction of mature osteoblast up to 15 dpf. As a result, larger mineralized opercular bone was observed in the synergy group at 15 dpf. Overall, B administered alone was not able to affect the intermediate or mature osteoblast populations and the effect was observed only in synergy with VD.

In conclusion, our study indicates that B may be able to potentiate the osteoblast-stimulating effect of VD or exert its pro-osteogenic effect in a VD-dependent manner. Our transcriptome analysis suggests that these effects could be through the induction of molecular programs involving the activation of MAPK and TGF-β/BMP pathways. These findings could lead to a promising approach in developing medicines to alleviate VD deficiency by synergically adding micronutrients along with VD to increase the overall efficiency of the treatment. In a world where improving bone health has become a matter of crucial importance, there is a high translational value for the findings of this study in the development of prophylactic measures focusing on improving VD supplementation efficiency in nutrition. Future studies should explore other outcomes of the combinatorial treatments such as its effects on stress or immunity to have a complete overview.

## Data Availability Statement

The datasets presented in this study can be found in online repositories. The names of the repository/repositories and accession number(s) can be found below: All the sequence data can be accessed through NCBI Bioproject - PRJNA796753.

## Ethics Statement

The experiment involving wildtype zebrafish were conducted in accordance with the Italian law on animal experimentation and were approved by the Ethics Committee of the Università Politecnica delle Marche, Ancona, Italy and by the Italian Ministry of Health (Aut. No. 583/2020-PR). All the experimental procedures involving transgenic zebrafish followed the EU Directive 2010/63/EU and Portuguese legislation - Decreto-Lei 113/2013 - for animal experimentation and welfare. Animal handling and experiments were performed by qualified operators accredited by the Portuguese Direção-Geral de Alimentação e Veterinária (DGAV) under authorization no. 012769/2021.

## Author Contributions

JS: conceptualization, methodology, data acquisition and analysis, writing—original draft, and writing—review and editing. MG: data analysis, methodology, and writing—review and editing. AC: data acquisition and analysis, methodology, and writing—review and editing. VG: data acquisition and analysis. PG: supervision and writing—review and editing. FM: conceptualization, supervision, and writing—review and editing. OC: conceptualization, supervision, writing—review and editing, and funding acquisition. All authors contributed to the article and approved the submitted version.

## Funding

This work is part of a project that has received funding from the European Union's Horizon 2020 Research and Innovation Program under the Marie Skłodowska-Curie Grant Agreement No. 766347 to OC and PG. Further funding was received through Portuguese national funds from FCT - Foundation for Science and Technology through projects UIDB/04326/2020, UIDP/04326/2020, and LA/P/0101/2020.

## Conflict of Interest

The authors declare that the research was conducted in the absence of any commercial or financial relationships that could be construed as a potential conflict of interest.

## Publisher's Note

All claims expressed in this article are solely those of the authors and do not necessarily represent those of their affiliated organizations, or those of the publisher, the editors and the reviewers. Any product that may be evaluated in this article, or claim that may be made by its manufacturer, is not guaranteed or endorsed by the publisher.
