# Supplementary material for: Zebrafish as a Model to Unveil the Pro-Osteogenic Effects of Boron-Vitamin D3 Synergism
Source: Front Nutr. 2022 Apr 29;9:868805. doi: 10.3389/fnut.2022.868805 (PMC9105455; doi:10.3389/fnut.2022.868805)
Supplement: Supplementary file 2 [file Data_Sheet_1.docx]

Supplementary Material

# Zebrafish as a model to unveil the pro-osteogenic effects of Boron-Vitamin D_3_ synergism

Jerry Maria Sojan^1^, Manu Kumar Gundappa^2^, Alessio Carletti^3,4^, Vasco Gaspar^4^, Paulo Gavaia^3,4^, Francesca Maradonna^1^ and Oliana Carnevali^1^^*^

^1^Department of Life and Environmental Sciences, Università Politecnica delle Marche, via Brecce Bianche, 60131 Ancona, Italy

^2^ The Roslin Institute and Royal (Dick) School of Veterinary Studies, The University of Edinburgh, Midlothian, United Kingdom

^3^ CCMAR, University of Algarve, Campus Gambelas, 8005-139 Faro, Portugal

^4^ Faculty of Medicine and Biomedical Sciences, University of Algarve, Campus de Gambelas, 8005-139 Faro, Portugal

^*^Correspondence: [o.carnevali@staff.univpm.it](mailto:o.carnevali@staff.univpm.it); Phone: +390712204990

**Supplementary Table 1 – Details of the data generated after sequencing, after initial QC across different groups.**

| **Sample** | **Raw reads (Pairs)** | **Sequence data** | **Q30 (%)** | **GC (%)** |
| --- | --- | --- | --- | --- |
| C-13 | 96708280 | 14,506,242,000 | 93.65 | 46.93 |
| B100-3 | 64455670 | 9,668,350,500 | 94.83 | 47.99 |
| VD-3 | 79895168 | 11,984,275,200 | 94.13 | 47.77 |
| VD-2 | 81963304 | 12,294,495,600 | 94.28 | 48.27 |
| B10VD-3 | 92865370 | 13,929,805,500 | 94.18 | 46.99 |
| B10-4 | 88668494 | 13,300,274,100 | 94.6 | 47.65 |
| C-2 | 102022422 | 15,303,363,300 | 94.22 | 48.1 |
| B10VD-1 | 104282710 | 15,642,406,500 | 93.81 | 48.15 |
| B100-1 | 71415618 | 10,712,342,700 | 94.54 | 48.33 |
| C-4 | 80591422 | 12,088,713,300 | 94.5 | 47.78 |
| B10-1 | 113506504 | 17,025,975,600 | 94.52 | 47.62 |
| B10-2 | 64377336 | 9,656,600,400 | 94.67 | 47.21 |
| VD-1 | 86162182 | 12,924,327,300 | 94.49 | 47.82 |
| B100VD-2 | 111101012 | 16,665,151,800 | 94.49 | 49.34 |
| B100-4 | 76966844 | 11,545,026,600 | 94.84 | 49.99 |
| VD-4 | 71121896 | 10,668,284,400 | 94.62 | 47.9 |
| B10-3 | 83129550 | 12,469,432,500 | 94.11 | 47.83 |
| B100VD-4 | 63256244 | 9,488,436,600 | 94.64 | 47.73 |
| B100VD-1 | 86462822 | 12,969,423,300 | 93.79 | 47.3 |
| C-1 | 84886368 | 12,732,955,200 | 94.35 | 47.01 |
| B10VD-4 | 86311546 | 12,946,731,900 | 94.68 | 47.88 |
| B100VD-3 | 88289786 | 13,243,467,900 | 94.18 | 47.5 |
| B100-2 | 91357382 | 13,703,607,300 | 94.29 | 47.73 |
| B10VD-2 | 96428740 | 14,464,311,000 | 94.2 | 48.78 |

Supplementary Table 2 - List of primers used in the Real-Time PCRs.

| Gene Acronym | NCBI gene accession no. | Forward | Reverse |
| --- | --- | --- | --- |
| *mapk14b* | NM_001313759.1 | CCAAGAGGAACTTCGCAGAC | GATCCAGCAGCTTTCAGGAC |
| *egfra* | NM_194424.1 | GGACGACCGCATGCATTTAC | AGGCTGAAAGTCTCCCTCCT |
| *ppp3cca* | NM_001166628.1 | ACAGAATGCCATCCAAGGCT | TCTTCTCGTTGGCAGCGTTA |
| *dusp2* | NM_001003451.1 | CGCAACGTCAACTGGAACTC | GGTTCTGATTGGAGTCGAGGC |
| *cacnb3b* | XM_682310.7 | CCTTTACACCTCAGGACCACC | CTGAGGACCCCCTGAGAAAC |
| *rps6ka3b* | NM_001083026.2 | AGTGAAGGTGTATGATGATGGC | GCCACAGTTTTGGTGATGGT |
| *mrasb* | XM_003200999.5 | CGAGTGCGAAAGATCCACCA | TAGAGGGTTTGCGCTCACAG |
| *hspb1* | NM_001008615.2 | GAGTTACGGACGAGCCCTTT | AGCACCCCGTCTTTGGTTTTA |
| *rpl13a* | NM_212784.1 | TCTGGAGGACTGTAAGAGGTATGC | AGACGCACAATCTTGAGAGCAG |
| *rplp0* | NM_131580.2 | CTGAACATCTCGCCCTTCTC | TAGCCGATCTGCAGACACAC |

**Supplementary Table 4 – GO enrichment results across genes differentially expressed in the three clusters.**

| Cluster | Go Category | ID | Description | GeneRatio | pvalue | p.adjust | qvalue | Count |
| --- | --- | --- | --- | --- | --- | --- | --- | --- |
| 1 | Biological Process | GO:0019941 | modification-dependent protein catabolic process | 90/1987 | 1.44E-09 | 1.66E-06 | 1.62E-06 | 90 |
| 1 | Biological Process | GO:0010499 | proteasomal ubiquitin-independent protein catabolic process | 16/1987 | 1.53E-09 | 1.66E-06 | 1.62E-06 | 16 |
| 1 | Biological Process | GO:0043632 | modification-dependent macromolecule catabolic process | 91/1987 | 1.63E-09 | 1.66E-06 | 1.62E-06 | 91 |
| 1 | Biological Process | GO:0006511 | ubiquitin-dependent protein catabolic process | 88/1987 | 2.24E-09 | 1.71E-06 | 1.67E-06 | 88 |
| 1 | Biological Process | GO:0043248 | proteasome assembly | 12/1987 | 1.54E-08 | 9.43E-06 | 9.2E-06 | 12 |
| 1 | Biological Process | GO:0002088 | lens development in camera-type eye | 30/1987 | 1.67E-07 | 8.54E-05 | 8.34E-05 | 30 |
| 1 | Biological Process | GO:0043161 | proteasome-mediated ubiquitin-dependent protein catabolic process | 58/1987 | 1.24E-06 | 0.000544 | 0.000531 | 58 |
| 1 | Biological Process | GO:0010498 | proteasomal protein catabolic process | 59/1987 | 5.06E-06 | 0.00194 | 0.001893 | 59 |
| 1 | Biological Process | GO:0043010 | camera-type eye development | 76/1987 | 7.95E-06 | 0.002709 | 0.002643 | 76 |
| 1 | Biological Process | GO:0006457 | protein folding | 33/1987 | 3.49E-05 | 0.010693 | 0.010433 | 33 |
| 1 | Biological Process | GO:0006081 | cellular aldehyde metabolic process | 14/1987 | 5.09E-05 | 0.014189 | 0.013844 | 14 |
| 1 | Biological Process | GO:0051149 | positive regulation of muscle cell differentiation | 7/1987 | 6.32E-05 | 0.014912 | 0.01455 | 7 |
| 1 | Biological Process | GO:0051155 | positive regulation of striated muscle cell differentiation | 7/1987 | 6.32E-05 | 0.014912 | 0.01455 | 7 |
| 1 | Biological Process | GO:0009408 | response to heat | 13/1987 | 9.41E-05 | 0.020593 | 0.020092 | 13 |
| 1 | Biological Process | GO:0016202 | regulation of striated muscle tissue development | 10/1987 | 0.000106 | 0.021745 | 0.021217 | 10 |
| 1 | Biological Process | GO:0050953 | sensory perception of light stimulus | 37/1987 | 0.00021 | 0.040213 | 0.039236 | 37 |
| 1 | Biological Process | GO:1901861 | regulation of muscle tissue development | 10/1987 | 0.000226 | 0.040777 | 0.039786 | 10 |
| 2 | Biological Process | GO:0006836 | neurotransmitter transport | 27/1240 | 5.18E-09 | 1.39E-05 | 1.28E-05 | 27 |
| 2 | Biological Process | GO:0018298 | protein-chromophore linkage | 14/1240 | 2.8E-07 | 0.000315 | 0.000291 | 14 |
| 2 | Biological Process | GO:0007602 | phototransduction | 15/1240 | 3.54E-07 | 0.000315 | 0.000291 | 15 |
| 2 | Biological Process | GO:0009583 | detection of light stimulus | 16/1240 | 1.39E-06 | 0.000927 | 0.000854 | 16 |
| 2 | Biological Process | GO:0043269 | regulation of ion transport | 38/1240 | 2.74E-06 | 0.001466 | 0.001352 | 38 |
| 2 | Biological Process | GO:0015893 | drug transport | 21/1240 | 4.97E-06 | 0.002216 | 0.002042 | 21 |
| 2 | Biological Process | GO:0009416 | response to light stimulus | 27/1240 | 1.06E-05 | 0.00332 | 0.00306 | 27 |
| 2 | Biological Process | GO:0009581 | detection of external stimulus | 18/1240 | 1.12E-05 | 0.00332 | 0.00306 | 18 |
| 2 | Biological Process | GO:0009582 | detection of abiotic stimulus | 18/1240 | 1.12E-05 | 0.00332 | 0.00306 | 18 |
| 2 | Biological Process | GO:0071482 | cellular response to light stimulus | 14/1240 | 1.79E-05 | 0.004356 | 0.004014 | 14 |
| 2 | Biological Process | GO:0001505 | regulation of neurotransmitter levels | 24/1240 | 1.95E-05 | 0.004356 | 0.004014 | 24 |
| 2 | Biological Process | GO:0051606 | detection of stimulus | 19/1240 | 1.95E-05 | 0.004356 | 0.004014 | 19 |
| 2 | Biological Process | GO:0050953 | sensory perception of light stimulus | 29/1240 | 2.32E-05 | 0.004781 | 0.004406 | 29 |
| 2 | Biological Process | GO:0034762 | regulation of transmembrane transport | 31/1240 | 4.32E-05 | 0.008253 | 0.007606 | 31 |
| 2 | Biological Process | GO:0007601 | visual perception | 27/1240 | 6.72E-05 | 0.011989 | 0.011049 | 27 |
| 2 | Biological Process | GO:0015672 | monovalent inorganic cation transport | 40/1240 | 8.05E-05 | 0.013467 | 0.012411 | 40 |
| 2 | Biological Process | GO:0015807 | L-amino acid transport | 8/1240 | 0.000121 | 0.019019 | 0.017529 | 8 |
| 2 | Biological Process | GO:0016079 | synaptic vesicle exocytosis | 13/1240 | 0.000139 | 0.01927 | 0.01776 | 13 |
| 2 | Biological Process | GO:0007269 | neurotransmitter secretion | 15/1240 | 0.000144 | 0.01927 | 0.01776 | 15 |
| 2 | Biological Process | GO:0099643 | signal release from synapse | 15/1240 | 0.000144 | 0.01927 | 0.01776 | 15 |
| 2 | Biological Process | GO:0034765 | regulation of ion transmembrane transport | 29/1240 | 0.000165 | 0.020984 | 0.01934 | 29 |
| 2 | Biological Process | GO:0045055 | regulated exocytosis | 16/1240 | 0.000196 | 0.0228 | 0.021013 | 16 |
| 2 | Biological Process | GO:0003333 | amino acid transmembrane transport | 11/1240 | 0.000196 | 0.0228 | 0.021013 | 11 |
| 2 | Biological Process | GO:0055003 | cardiac myofibril assembly | 9/1240 | 0.000295 | 0.032902 | 0.030324 | 9 |
| 2 | Biological Process | GO:0006865 | amino acid transport | 13/1240 | 0.000314 | 0.033658 | 0.031021 | 13 |
| 2 | Biological Process | GO:0071478 | cellular response to radiation | 14/1240 | 0.00033 | 0.033966 | 0.031304 | 14 |
| 2 | Biological Process | GO:0071214 | cellular response to abiotic stimulus | 15/1240 | 0.00038 | 0.036355 | 0.033506 | 15 |
| 2 | Biological Process | GO:0104004 | cellular response to environmental stimulus | 15/1240 | 0.00038 | 0.036355 | 0.033506 | 15 |
| 2 | Biological Process | GO:0098655 | cation transmembrane transport | 51/1240 | 0.000414 | 0.038105 | 0.03512 | 51 |
| 2 | Biological Process | GO:0015850 | organic hydroxy compound transport | 15/1240 | 0.000433 | 0.038105 | 0.03512 | 15 |
| 2 | Biological Process | GO:0009314 | response to radiation | 28/1240 | 0.000441 | 0.038105 | 0.03512 | 28 |
| 2 | Biological Process | GO:0051260 | protein homooligomerization | 21/1240 | 0.000471 | 0.038555 | 0.035534 | 21 |
| 2 | Biological Process | GO:0098656 | anion transmembrane transport | 20/1240 | 0.000475 | 0.038555 | 0.035534 | 20 |
| 2 | Biological Process | GO:1901879 | regulation of protein depolymerization | 11/1240 | 0.000491 | 0.038638 | 0.03561 | 11 |
| 2 | Biological Process | GO:0006936 | muscle contraction | 21/1240 | 0.000516 | 0.039487 | 0.036392 | 21 |
| 2 | Biological Process | GO:0006353 | DNA-templated transcription, termination | 5/1240 | 0.000608 | 0.043529 | 0.040118 | 5 |
| 2 | Biological Process | GO:0048739 | cardiac muscle fiber development | 7/1240 | 0.000611 | 0.043529 | 0.040118 | 7 |
| 2 | Biological Process | GO:0015844 | monoamine transport | 8/1240 | 0.000622 | 0.043529 | 0.040118 | 8 |
| 2 | Biological Process | GO:1903825 | organic acid transmembrane transport | 13/1240 | 0.000651 | 0.043529 | 0.040118 | 13 |
| 2 | Biological Process | GO:1905039 | carboxylic acid transmembrane transport | 13/1240 | 0.000651 | 0.043529 | 0.040118 | 13 |
| 2 | Biological Process | GO:0043244 | regulation of protein-containing complex disassembly | 12/1240 | 0.000731 | 0.047637 | 0.043904 | 12 |
| 2 | Biological Process | GO:1902903 | regulation of supramolecular fiber organization | 22/1240 | 0.000776 | 0.047637 | 0.043904 | 22 |
| 2 | Biological Process | GO:0014046 | dopamine secretion | 7/1240 | 0.000801 | 0.047637 | 0.043904 | 7 |
| 2 | Biological Process | GO:0014059 | regulation of dopamine secretion | 7/1240 | 0.000801 | 0.047637 | 0.043904 | 7 |
| 2 | Biological Process | GO:0030241 | skeletal muscle myosin thick filament assembly | 7/1240 | 0.000801 | 0.047637 | 0.043904 | 7 |
| 2 | Biological Process | GO:0006820 | anion transport | 42/1240 | 0.000839 | 0.048803 | 0.044979 | 42 |
| 3 | Biological Process | GO:0030198 | extracellular matrix organization | 51/1501 | 9.03E-20 | 1.3E-16 | 1.22E-16 | 51 |
| 3 | Biological Process | GO:0043062 | extracellular structure organization | 51/1501 | 9.03E-20 | 1.3E-16 | 1.22E-16 | 51 |
| 3 | Biological Process | GO:0030199 | collagen fibril organization | 12/1501 | 3.08E-08 | 2.47E-05 | 2.33E-05 | 12 |
| 3 | Biological Process | GO:0007229 | integrin-mediated signaling pathway | 20/1501 | 3.44E-08 | 2.47E-05 | 2.33E-05 | 20 |
| 3 | Biological Process | GO:0031589 | cell-substrate adhesion | 24/1501 | 8.48E-08 | 4.88E-05 | 4.59E-05 | 24 |
| 3 | Biological Process | GO:0061448 | connective tissue development | 36/1501 | 6.76E-07 | 0.000276 | 0.000259 | 36 |
| 3 | Biological Process | GO:0090171 | chondrocyte morphogenesis | 10/1501 | 7.55E-07 | 0.000276 | 0.000259 | 10 |
| 3 | Biological Process | GO:0006820 | anion transport | 59/1501 | 7.66E-07 | 0.000276 | 0.000259 | 59 |
| 3 | Biological Process | GO:0001501 | skeletal system development | 61/1501 | 1.58E-06 | 0.000504 | 0.000474 | 61 |
| 3 | Biological Process | GO:0003414 | chondrocyte morphogenesis involved in endochondral bone morphogenesis | 9/1501 | 2.76E-06 | 0.00062 | 0.000583 | 9 |
| 3 | Biological Process | GO:0003429 | growth plate cartilage chondrocyte morphogenesis | 9/1501 | 2.76E-06 | 0.00062 | 0.000583 | 9 |
| 3 | Biological Process | GO:0003433 | chondrocyte development involved in endochondral bone morphogenesis | 9/1501 | 2.76E-06 | 0.00062 | 0.000583 | 9 |
| 3 | Biological Process | GO:0051216 | cartilage development | 34/1501 | 2.8E-06 | 0.00062 | 0.000583 | 34 |
| 3 | Biological Process | GO:0015711 | organic anion transport | 44/1501 | 4.41E-06 | 0.000826 | 0.000777 | 44 |
| 3 | Biological Process | GO:0031101 | fin regeneration | 21/1501 | 4.61E-06 | 0.000826 | 0.000777 | 21 |
| 3 | Biological Process | GO:0003413 | chondrocyte differentiation involved in endochondral bone morphogenesis | 9/1501 | 4.88E-06 | 0.000826 | 0.000777 | 9 |
| 3 | Biological Process | GO:0003418 | growth plate cartilage chondrocyte differentiation | 9/1501 | 4.88E-06 | 0.000826 | 0.000777 | 9 |
| 3 | Biological Process | GO:0002062 | chondrocyte differentiation | 14/1501 | 6.81E-06 | 0.001088 | 0.001023 | 14 |
| 3 | Biological Process | GO:0002063 | chondrocyte development | 11/1501 | 1.29E-05 | 0.001934 | 0.001818 | 11 |
| 3 | Biological Process | GO:0003422 | growth plate cartilage morphogenesis | 9/1501 | 1.34E-05 | 0.001934 | 0.001818 | 9 |
| 3 | Biological Process | GO:0060536 | cartilage morphogenesis | 13/1501 | 2.7E-05 | 0.003701 | 0.00348 | 13 |
| 3 | Biological Process | GO:0003416 | endochondral bone growth | 9/1501 | 3.24E-05 | 0.004048 | 0.003807 | 9 |
| 3 | Biological Process | GO:0003417 | growth plate cartilage development | 9/1501 | 3.24E-05 | 0.004048 | 0.003807 | 9 |
| 3 | Biological Process | GO:0018158 | protein oxidation | 6/1501 | 3.66E-05 | 0.004383 | 0.004121 | 6 |
| 3 | Biological Process | GO:0097435 | supramolecular fiber organization | 63/1501 | 4.1E-05 | 0.004706 | 0.004425 | 63 |
| 3 | Biological Process | GO:0006270 | DNA replication initiation | 10/1501 | 4.25E-05 | 0.004706 | 0.004425 | 10 |
| 3 | Biological Process | GO:0098868 | bone growth | 9/1501 | 4.82E-05 | 0.005135 | 0.004828 | 9 |
| 3 | Biological Process | GO:0015849 | organic acid transport | 28/1501 | 7.31E-05 | 0.007506 | 0.007058 | 28 |
| 3 | Biological Process | GO:0007160 | cell-matrix adhesion | 14/1501 | 7.84E-05 | 0.00778 | 0.007316 | 14 |
| 3 | Biological Process | GO:0048514 | blood vessel morphogenesis | 58/1501 | 8.3E-05 | 0.007955 | 0.00748 | 58 |
| 3 | Biological Process | GO:0040007 | growth | 58/1501 | 0.000144 | 0.013292 | 0.012499 | 58 |
| 3 | Biological Process | GO:0046942 | carboxylic acid transport | 27/1501 | 0.000148 | 0.013292 | 0.012499 | 27 |
| 3 | Biological Process | GO:0009611 | response to wounding | 42/1501 | 0.00016 | 0.01399 | 0.013154 | 42 |
| 3 | Biological Process | GO:0033627 | cell adhesion mediated by integrin | 7/1501 | 0.000173 | 0.014597 | 0.013725 | 7 |
| 3 | Biological Process | GO:0060350 | endochondral bone morphogenesis | 10/1501 | 0.000253 | 0.02061 | 0.01938 | 10 |
| 3 | Biological Process | GO:0060351 | cartilage development involved in endochondral bone morphogenesis | 9/1501 | 0.000258 | 0.02061 | 0.01938 | 9 |
| 3 | Biological Process | GO:0042060 | wound healing | 35/1501 | 0.000338 | 0.026313 | 0.024743 | 35 |
| 3 | Biological Process | GO:0090504 | epiboly | 17/1501 | 0.000486 | 0.035997 | 0.033848 | 17 |
| 3 | Biological Process | GO:0048589 | developmental growth | 53/1501 | 0.000488 | 0.035997 | 0.033848 | 53 |
| 3 | Biological Process | GO:0010810 | regulation of cell-substrate adhesion | 8/1501 | 0.000606 | 0.042405 | 0.039874 | 8 |
| 3 | Biological Process | GO:0010811 | positive regulation of cell-substrate adhesion | 6/1501 | 0.000618 | 0.042405 | 0.039874 | 6 |
| 3 | Biological Process | GO:0030903 | notochord development | 15/1501 | 0.000619 | 0.042405 | 0.039874 | 15 |
| 3 | Biological Process | GO:0002011 | morphogenesis of an epithelial sheet | 19/1501 | 0.000684 | 0.045687 | 0.04296 | 19 |
| 3 | Biological Process | GO:0001525 | angiogenesis | 46/1501 | 0.000699 | 0.045687 | 0.04296 | 46 |
| 1 | Cellular Component | GO:0000502 | proteasome complex | 42/1966 | 4.03E-23 | 9.95E-21 | 9.75E-21 | 42 |
| 1 | Cellular Component | GO:1905369 | endopeptidase complex | 42/1966 | 4.03E-23 | 9.95E-21 | 9.75E-21 | 42 |
| 1 | Cellular Component | GO:0005838 | proteasome regulatory particle | 23/1966 | 7.19E-19 | 1.18E-16 | 1.16E-16 | 23 |
| 1 | Cellular Component | GO:1905368 | peptidase complex | 42/1966 | 5.61E-18 | 6.93E-16 | 6.79E-16 | 42 |
| 1 | Cellular Component | GO:0022624 | proteasome accessory complex | 23/1966 | 4.09E-16 | 4.04E-14 | 3.96E-14 | 23 |
| 1 | Cellular Component | GO:0005839 | proteasome core complex | 16/1966 | 9.8E-11 | 8.07E-09 | 7.91E-09 | 16 |
| 1 | Cellular Component | GO:0008540 | proteasome regulatory particle, base subcomplex | 12/1966 | 1.4E-09 | 9.91E-08 | 9.71E-08 | 12 |
| 1 | Cellular Component | GO:0008541 | proteasome regulatory particle, lid subcomplex | 9/1966 | 1.01E-08 | 6.23E-07 | 6.11E-07 | 9 |
| 1 | Cellular Component | GO:0019773 | proteasome core complex, alpha-subunit complex | 9/1966 | 5.05E-08 | 2.77E-06 | 2.71E-06 | 9 |
| 1 | Cellular Component | GO:0000323 | lytic vacuole | 35/1966 | 0.000641 | 0.027382 | 0.02684 | 35 |
| 1 | Cellular Component | GO:0005764 | lysosome | 35/1966 | 0.000641 | 0.027382 | 0.02684 | 35 |
| 1 | Cellular Component | GO:0005765 | lysosomal membrane | 19/1966 | 0.000721 | 0.027382 | 0.02684 | 19 |
| 1 | Cellular Component | GO:0098852 | lytic vacuole membrane | 19/1966 | 0.000721 | 0.027382 | 0.02684 | 19 |
| 2 | Cellular Component | GO:0070382 | exocytic vesicle | 23/1275 | 2.01E-05 | 0.003069 | 0.002651 | 23 |
| 2 | Cellular Component | GO:0005865 | striated muscle thin filament | 14/1275 | 2.6E-05 | 0.003069 | 0.002651 | 14 |
| 2 | Cellular Component | GO:0036379 | myofilament | 14/1275 | 2.6E-05 | 0.003069 | 0.002651 | 14 |
| 2 | Cellular Component | GO:0099503 | secretory vesicle | 26/1275 | 3.15E-05 | 0.003069 | 0.002651 | 26 |
| 2 | Cellular Component | GO:0030017 | sarcomere | 24/1275 | 4.4E-05 | 0.003069 | 0.002651 | 24 |
| 2 | Cellular Component | GO:0044449 | contractile fiber part | 24/1275 | 4.92E-05 | 0.003069 | 0.002651 | 24 |
| 2 | Cellular Component | GO:0045202 | synapse | 54/1275 | 5.93E-05 | 0.003069 | 0.002651 | 54 |
| 2 | Cellular Component | GO:0030016 | myofibril | 24/1275 | 6.1E-05 | 0.003069 | 0.002651 | 24 |
| 2 | Cellular Component | GO:0043292 | contractile fiber | 24/1275 | 6.79E-05 | 0.003069 | 0.002651 | 24 |
| 2 | Cellular Component | GO:0097731 | 9+0 non-motile cilium | 11/1275 | 0.000183 | 0.006678 | 0.005769 | 11 |
| 2 | Cellular Component | GO:0097733 | photoreceptor cell cilium | 11/1275 | 0.000183 | 0.006678 | 0.005769 | 11 |
| 2 | Cellular Component | GO:0030018 | Z disc | 15/1275 | 0.000197 | 0.006678 | 0.005769 | 15 |
| 2 | Cellular Component | GO:0031674 | I band | 16/1275 | 0.000302 | 0.009403 | 0.008122 | 16 |
| 2 | Cellular Component | GO:0031430 | M band | 8/1275 | 0.000323 | 0.009403 | 0.008122 | 8 |
| 2 | Cellular Component | GO:0008021 | synaptic vesicle | 18/1275 | 0.000402 | 0.010334 | 0.008927 | 18 |
| 2 | Cellular Component | GO:0015629 | actin cytoskeleton | 38/1275 | 0.000406 | 0.010334 | 0.008927 | 38 |
| 2 | Cellular Component | GO:0098793 | presynapse | 24/1275 | 0.000453 | 0.010847 | 0.00937 | 24 |
| 2 | Cellular Component | GO:0034703 | cation channel complex | 24/1275 | 0.000493 | 0.011068 | 0.009561 | 24 |
| 2 | Cellular Component | GO:0001750 | photoreceptor outer segment | 9/1275 | 0.000517 | 0.011068 | 0.009561 | 9 |
| 2 | Cellular Component | GO:0044456 | synapse part | 42/1275 | 0.000638 | 0.012978 | 0.011211 | 42 |
| 2 | Cellular Component | GO:0097730 | non-motile cilium | 12/1275 | 0.000722 | 0.013985 | 0.01208 | 12 |
| 2 | Cellular Component | GO:0005929 | cilium | 34/1275 | 0.000808 | 0.014954 | 0.012918 | 34 |
| 2 | Cellular Component | GO:0030133 | transport vesicle | 25/1275 | 0.001037 | 0.018352 | 0.015853 | 25 |
| 2 | Cellular Component | GO:0031672 | A band | 8/1275 | 0.001328 | 0.021892 | 0.018911 | 8 |
| 2 | Cellular Component | GO:0044463 | cell projection part | 49/1275 | 0.001406 | 0.021892 | 0.018911 | 49 |
| 2 | Cellular Component | GO:0120038 | plasma membrane bounded cell projection part | 49/1275 | 0.001406 | 0.021892 | 0.018911 | 49 |
| 2 | Cellular Component | GO:0008076 | voltage-gated potassium channel complex | 10/1275 | 0.00156 | 0.021892 | 0.018911 | 10 |
| 2 | Cellular Component | GO:0030672 | synaptic vesicle membrane | 10/1275 | 0.00156 | 0.021892 | 0.018911 | 10 |
| 2 | Cellular Component | GO:0099501 | exocytic vesicle membrane | 10/1275 | 0.00156 | 0.021892 | 0.018911 | 10 |
| 2 | Cellular Component | GO:0044441 | ciliary part | 26/1275 | 0.00208 | 0.028223 | 0.02438 | 26 |
| 2 | Cellular Component | GO:0098797 | plasma membrane protein complex | 48/1275 | 0.002477 | 0.032523 | 0.028094 | 48 |
| 2 | Cellular Component | GO:0001518 | voltage-gated sodium channel complex | 5/1275 | 0.002884 | 0.036687 | 0.031691 | 5 |
| 2 | Cellular Component | GO:1902495 | transmembrane transporter complex | 29/1275 | 0.003341 | 0.041209 | 0.035598 | 29 |
| 2 | Cellular Component | GO:0034702 | ion channel complex | 28/1275 | 0.00345 | 0.041299 | 0.035675 | 28 |
| 3 | Cellular Component | GO:0031012 | extracellular matrix | 85/1543 | 5.84E-33 | 2.52E-30 | 2.38E-30 | 85 |
| 3 | Cellular Component | GO:0062023 | collagen-containing extracellular matrix | 41/1543 | 6.32E-20 | 1.37E-17 | 1.29E-17 | 41 |
| 3 | Cellular Component | GO:0005581 | collagen trimer | 32/1543 | 2.6E-16 | 3.75E-14 | 3.53E-14 | 32 |
| 3 | Cellular Component | GO:0008305 | integrin complex | 17/1543 | 1.07E-09 | 9.28E-08 | 8.75E-08 | 17 |
| 3 | Cellular Component | GO:0098636 | protein complex involved in cell adhesion | 17/1543 | 1.07E-09 | 9.28E-08 | 8.75E-08 | 17 |
| 3 | Cellular Component | GO:0044420 | extracellular matrix component | 10/1543 | 1.54E-06 | 0.000111 | 0.000104 | 10 |
| 3 | Cellular Component | GO:0098644 | complex of collagen trimers | 7/1543 | 1.93E-06 | 0.000119 | 0.000112 | 7 |
| 3 | Cellular Component | GO:0042555 | MCM complex | 8/1543 | 1.99E-05 | 0.001076 | 0.001014 | 8 |
| 3 | Cellular Component | GO:0005925 | focal adhesion | 14/1543 | 0.000138 | 0.006614 | 0.006237 | 14 |
| 3 | Cellular Component | GO:0005924 | cell-substrate adherens junction | 14/1543 | 0.000168 | 0.006614 | 0.006237 | 14 |
| 3 | Cellular Component | GO:0030055 | cell-substrate junction | 14/1543 | 0.000168 | 0.006614 | 0.006237 | 14 |
| 3 | Cellular Component | GO:0005732 | small nucleolar ribonucleoprotein complex | 7/1543 | 0.000426 | 0.015333 | 0.014459 | 7 |
| 3 | Cellular Component | GO:0005882 | intermediate filament | 17/1543 | 0.000752 | 0.024984 | 0.02356 | 17 |
| 3 | Cellular Component | GO:0045111 | intermediate filament cytoskeleton | 17/1543 | 0.000855 | 0.026388 | 0.024883 | 17 |
| 3 | Cellular Component | GO:0030054 | cell junction | 55/1543 | 0.000943 | 0.027165 | 0.025616 | 55 |
| 3 | Cellular Component | GO:0005923 | bicellular tight junction | 15/1543 | 0.001266 | 0.034193 | 0.032243 | 15 |
| 3 | Cellular Component | GO:0045178 | basal part of cell | 6/1543 | 0.001442 | 0.03664 | 0.034551 | 6 |
| 3 | Cellular Component | GO:0070160 | tight junction | 15/1543 | 0.001647 | 0.039525 | 0.037272 | 15 |
| 1 | Molecular Function | GO:0004298 | threonine-type endopeptidase activity | 16/1989 | 2.4E-10 | 1.02E-07 | 9.75E-08 | 16 |
| 1 | Molecular Function | GO:0070003 | threonine-type peptidase activity | 16/1989 | 2.4E-10 | 1.02E-07 | 9.75E-08 | 16 |
| 1 | Molecular Function | GO:0004175 | endopeptidase activity | 83/1989 | 1.27E-06 | 0.000361 | 0.000344 | 83 |
| 1 | Molecular Function | GO:0016798 | hydrolase activity, acting on glycosyl bonds | 32/1989 | 1.39E-05 | 0.002954 | 0.002818 | 32 |
| 1 | Molecular Function | GO:0005212 | structural constituent of eye lens | 20/1989 | 4.12E-05 | 0.007016 | 0.006692 | 20 |
| 1 | Molecular Function | GO:0051787 | misfolded protein binding | 10/1989 | 5.89E-05 | 0.008364 | 0.007977 | 10 |
| 1 | Molecular Function | GO:0000977 | RNA polymerase II regulatory region sequence-specific DNA binding | 78/1989 | 7.6E-05 | 0.009254 | 0.008827 | 78 |
| 1 | Molecular Function | GO:0019842 | vitamin binding | 26/1989 | 0.00015 | 0.016006 | 0.015266 | 26 |
| 1 | Molecular Function | GO:0005506 | iron ion binding | 41/1989 | 0.000232 | 0.021975 | 0.02096 | 41 |
| 1 | Molecular Function | GO:0001228 | DNA-binding transcription activator activity, RNA polymerase II-specific | 23/1989 | 0.000298 | 0.025369 | 0.024197 | 23 |
| 1 | Molecular Function | GO:0008237 | metallopeptidase activity | 36/1989 | 0.000328 | 0.025369 | 0.024197 | 36 |
| 1 | Molecular Function | GO:0004553 | hydrolase activity, hydrolyzing O-glycosyl compounds | 25/1989 | 0.00043 | 0.030559 | 0.029147 | 25 |
| 1 | Molecular Function | GO:0046906 | tetrapyrrole binding | 35/1989 | 0.000673 | 0.044085 | 0.042048 | 35 |
| 1 | Molecular Function | GO:0020037 | heme binding | 34/1989 | 0.000926 | 0.049895 | 0.047589 | 34 |
| 1 | Molecular Function | GO:0008236 | serine-type peptidase activity | 38/1989 | 0.000937 | 0.049895 | 0.047589 | 38 |
| 1 | Molecular Function | GO:0017171 | serine hydrolase activity | 38/1989 | 0.000937 | 0.049895 | 0.047589 | 38 |
| 2 | Molecular Function | GO:0009881 | photoreceptor activity | 16/1293 | 5.01E-08 | 3.77E-05 | 3.42E-05 | 16 |
| 2 | Molecular Function | GO:0005326 | neurotransmitter transporter activity | 17/1293 | 5.53E-07 | 0.000208 | 0.000189 | 17 |
| 2 | Molecular Function | GO:0008307 | structural constituent of muscle | 11/1293 | 1.14E-06 | 0.000249 | 0.000226 | 11 |
| 2 | Molecular Function | GO:0008020 | G protein-coupled photoreceptor activity | 13/1293 | 1.32E-06 | 0.000249 | 0.000226 | 13 |
| 2 | Molecular Function | GO:0046873 | metal ion transmembrane transporter activity | 59/1293 | 4.96E-06 | 0.000747 | 0.000678 | 59 |
| 2 | Molecular Function | GO:0015171 | amino acid transmembrane transporter activity | 18/1293 | 1.05E-05 | 0.001319 | 0.001197 | 18 |
| 2 | Molecular Function | GO:0008509 | anion transmembrane transporter activity | 41/1293 | 3.86E-05 | 0.004154 | 0.003769 | 41 |
| 2 | Molecular Function | GO:0017080 | sodium channel regulator activity | 6/1293 | 5.37E-05 | 0.004234 | 0.003841 | 6 |
| 2 | Molecular Function | GO:0022839 | ion gated channel activity | 48/1293 | 5.4E-05 | 0.004234 | 0.003841 | 48 |
| 2 | Molecular Function | GO:0022836 | gated channel activity | 49/1293 | 6.01E-05 | 0.004234 | 0.003841 | 49 |
| 2 | Molecular Function | GO:0046943 | carboxylic acid transmembrane transporter activity | 25/1293 | 6.18E-05 | 0.004234 | 0.003841 | 25 |
| 2 | Molecular Function | GO:0005342 | organic acid transmembrane transporter activity | 25/1293 | 7.59E-05 | 0.004763 | 0.004321 | 25 |
| 2 | Molecular Function | GO:0005328 | neurotransmitter:sodium symporter activity | 11/1293 | 0.000101 | 0.005778 | 0.005242 | 11 |
| 2 | Molecular Function | GO:0005244 | voltage-gated ion channel activity | 30/1293 | 0.000107 | 0.005778 | 0.005242 | 30 |
| 2 | Molecular Function | GO:0005216 | ion channel activity | 55/1293 | 0.000132 | 0.006646 | 0.00603 | 55 |
| 2 | Molecular Function | GO:0022832 | voltage-gated channel activity | 30/1293 | 0.000162 | 0.007622 | 0.006915 | 30 |
| 2 | Molecular Function | GO:0015077 | monovalent inorganic cation transmembrane transporter activity | 50/1293 | 0.000175 | 0.00774 | 0.007022 | 50 |
| 2 | Molecular Function | GO:0015179 | L-amino acid transmembrane transporter activity | 11/1293 | 0.000234 | 0.009261 | 0.008402 | 11 |
| 2 | Molecular Function | GO:0099106 | ion channel regulator activity | 11/1293 | 0.000234 | 0.009261 | 0.008402 | 11 |
| 2 | Molecular Function | GO:0022843 | voltage-gated cation channel activity | 25/1293 | 0.00026 | 0.00967 | 0.008773 | 25 |
| 2 | Molecular Function | GO:0051015 | actin filament binding | 30/1293 | 0.00028 | 0.00967 | 0.008773 | 30 |
| 2 | Molecular Function | GO:0008514 | organic anion transmembrane transporter activity | 28/1293 | 0.000283 | 0.00967 | 0.008773 | 28 |
| 2 | Molecular Function | GO:0015081 | sodium ion transmembrane transporter activity | 24/1293 | 0.000344 | 0.010952 | 0.009936 | 24 |
| 2 | Molecular Function | GO:0005267 | potassium channel activity | 22/1293 | 0.000349 | 0.010952 | 0.009936 | 22 |
| 2 | Molecular Function | GO:0015238 | drug transmembrane transporter activity | 14/1293 | 0.000465 | 0.013801 | 0.01252 | 14 |
| 2 | Molecular Function | GO:0005249 | voltage-gated potassium channel activity | 18/1293 | 0.000477 | 0.013801 | 0.01252 | 18 |
| 2 | Molecular Function | GO:0016247 | channel regulator activity | 12/1293 | 0.000556 | 0.015514 | 0.014075 | 12 |
| 2 | Molecular Function | GO:0042805 | actinin binding | 9/1293 | 0.000662 | 0.01779 | 0.01614 | 9 |
| 2 | Molecular Function | GO:0005261 | cation channel activity | 42/1293 | 0.000705 | 0.018306 | 0.016608 | 42 |
| 2 | Molecular Function | GO:0051371 | muscle alpha-actinin binding | 8/1293 | 0.001113 | 0.027895 | 0.025307 | 8 |
| 2 | Molecular Function | GO:0003779 | actin binding | 47/1293 | 0.001148 | 0.027895 | 0.025307 | 47 |
| 2 | Molecular Function | GO:0051393 | alpha-actinin binding | 8/1293 | 0.001372 | 0.032283 | 0.029288 | 8 |
| 2 | Molecular Function | GO:0005283 | amino acid:sodium symporter activity | 6/1293 | 0.001423 | 0.032481 | 0.029468 | 6 |
| 2 | Molecular Function | GO:0005313 | L-glutamate transmembrane transporter activity | 5/1293 | 0.00151 | 0.032484 | 0.029471 | 5 |
| 2 | Molecular Function | GO:0015172 | acidic amino acid transmembrane transporter activity | 5/1293 | 0.00151 | 0.032484 | 0.029471 | 5 |
| 2 | Molecular Function | GO:0015079 | potassium ion transmembrane transporter activity | 25/1293 | 0.001709 | 0.035745 | 0.03243 | 25 |
| 2 | Molecular Function | GO:0015370 | solute:sodium symporter activity | 15/1293 | 0.001882 | 0.037304 | 0.033844 | 15 |
| 2 | Molecular Function | GO:0005416 | amino acid:cation symporter activity | 6/1293 | 0.001883 | 0.037304 | 0.033844 | 6 |
| 3 | Molecular Function | GO:0005201 | extracellular matrix structural constituent | 36/1495 | 1.82E-22 | 1.42E-19 | 1.3E-19 | 36 |
| 3 | Molecular Function | GO:0050839 | cell adhesion molecule binding | 24/1495 | 2.67E-06 | 0.000876 | 0.000801 | 24 |
| 3 | Molecular Function | GO:0008514 | organic anion transmembrane transporter activity | 36/1495 | 3.36E-06 | 0.000876 | 0.000801 | 36 |
| 3 | Molecular Function | GO:0015291 | secondary active transmembrane transporter activity | 41/1495 | 5.91E-06 | 0.001049 | 0.000959 | 41 |
| 3 | Molecular Function | GO:0005178 | integrin binding | 14/1495 | 6.7E-06 | 0.001049 | 0.000959 | 14 |
| 3 | Molecular Function | GO:0008509 | anion transmembrane transporter activity | 47/1495 | 1.23E-05 | 0.001599 | 0.001462 | 47 |
| 3 | Molecular Function | GO:0022804 | active transmembrane transporter activity | 56/1495 | 1.44E-05 | 0.001615 | 0.001477 | 56 |
| 3 | Molecular Function | GO:0003688 | DNA replication origin binding | 9/1495 | 3.93E-05 | 0.003848 | 0.003518 | 9 |
| 3 | Molecular Function | GO:0005342 | organic acid transmembrane transporter activity | 28/1495 | 4.67E-05 | 0.003866 | 0.003534 | 28 |
| 3 | Molecular Function | GO:0038024 | cargo receptor activity | 15/1495 | 4.94E-05 | 0.003866 | 0.003534 | 15 |
| 3 | Molecular Function | GO:0046943 | carboxylic acid transmembrane transporter activity | 27/1495 | 9.67E-05 | 0.006883 | 0.006292 | 27 |
| 3 | Molecular Function | GO:0015293 | symporter activity | 26/1495 | 0.000218 | 0.013789 | 0.012606 | 26 |
| 3 | Molecular Function | GO:0005539 | glycosaminoglycan binding | 20/1495 | 0.000229 | 0.013789 | 0.012606 | 20 |
| 3 | Molecular Function | GO:0005044 | scavenger receptor activity | 12/1495 | 0.000364 | 0.020345 | 0.018599 | 12 |
| 3 | Molecular Function | GO:0015106 | bicarbonate transmembrane transporter activity | 7/1495 | 0.000669 | 0.033112 | 0.03027 | 7 |
| 3 | Molecular Function | GO:0005540 | hyaluronic acid binding | 8/1495 | 0.000677 | 0.033112 | 0.03027 | 8 |
| 3 | Molecular Function | GO:0004222 | metalloendopeptidase activity | 19/1495 | 0.000991 | 0.045645 | 0.041727 | 19 |
| 3 | Molecular Function | GO:0015370 | solute:sodium symporter activity | 17/1495 | 0.001136 | 0.048203 | 0.044066 | 17 |
| 3 | Molecular Function | GO:0015081 | sodium ion transmembrane transporter activity | 25/1495 | 0.001174 | 0.048203 | 0.044066 | 25 |
| 3 | Molecular Function | GO:0005518 | collagen binding | 7/1495 | 0.001231 | 0.048203 | 0.044066 | 7 |

**Supplementary Table 5 – Results from KEGG pathway enrichment analysis performed across the DEGs against the three different clusters.**

| cluster | ID | Description | GeneRatio | BgRatio | pvalue | p.adjust | qvalue | Count |
| --- | --- | --- | --- | --- | --- | --- | --- | --- |
| 1 | dre03050 | Proteasome | 40/820 | 57/6867 | 9.62E-25 | 1.47E-22 | 1.3E-22 | 40 |
| 1 | dre00830 | Retinol metabolism | 34/820 | 91/6867 | 2.96E-10 | 2.27E-08 | 2E-08 | 34 |
| 1 | dre00980 | Metabolism of xenobiotics by cytochrome P450 | 28/820 | 77/6867 | 2.24E-08 | 1.14E-06 | 1.01E-06 | 28 |
| 1 | dre04141 | Protein processing in endoplasmic reticulum | 49/820 | 202/6867 | 6.03E-07 | 2.2E-05 | 1.93E-05 | 49 |
| 1 | dre00860 | Porphyrin and chlorophyll metabolism | 23/820 | 65/6867 | 7.18E-07 | 2.2E-05 | 1.93E-05 | 23 |
| 1 | dre00053 | Ascorbate and aldarate metabolism | 19/820 | 53/6867 | 5.33E-06 | 0.000118 | 0.000104 | 19 |
| 1 | dre00982 | Drug metabolism - cytochrome P450 | 23/820 | 72/6867 | 5.4E-06 | 0.000118 | 0.000104 | 23 |
| 1 | dre00040 | Pentose and glucuronate interconversions | 18/820 | 55/6867 | 3.92E-05 | 0.00075 | 0.000661 | 18 |
| 1 | dre00500 | Starch and sucrose metabolism | 14/820 | 38/6867 | 6.55E-05 | 0.001114 | 0.000981 | 14 |
| 1 | dre00140 | Steroid hormone biosynthesis | 22/820 | 78/6867 | 7.5E-05 | 0.001147 | 0.00101 | 22 |
| 1 | dre00052 | Galactose metabolism | 12/820 | 35/6867 | 0.000472 | 0.006559 | 0.005776 | 12 |
| 2 | dre04744 | Phototransduction | 21/492 | 49/6867 | 3.55E-12 | 4.97E-10 | 4.71E-10 | 21 |
| 3 | dre04510 | Focal adhesion | 77/654 | 278/6867 | 5.61E-19 | 7.79E-17 | 6.79E-17 | 77 |
| 3 | dre04512 | ECM-receptor interaction | 44/654 | 107/6867 | 2.2E-18 | 1.53E-16 | 1.33E-16 | 44 |
| 3 | dre04810 | Regulation of actin cytoskeleton | 55/654 | 303/6867 | 1.46E-06 | 6.75E-05 | 5.88E-05 | 55 |
| 3 | dre00240 | Pyrimidine metabolism | 19/654 | 73/6867 | 3.48E-05 | 0.00121 | 0.001054 | 19 |
| 3 | dre04514 | Cell adhesion molecules | 31/654 | 183/6867 | 0.001012 | 0.028126 | 0.024494 | 31 |

**\**

**Supplementary Figure 1 – Gap statistic method-based cluster determination**

**Supplementary Figure 2. KEGG pathway maps for key skeletal pathways generated using Pathview showing differentially expressed genes in: A) VD treatment in comparison to the control; B) B10VD treatment in comparison to the control; C) B100VD treatment in comparison to the control; Green colours indicated increased expression and red colour indicates decreased expression in the selected group compared to control.**


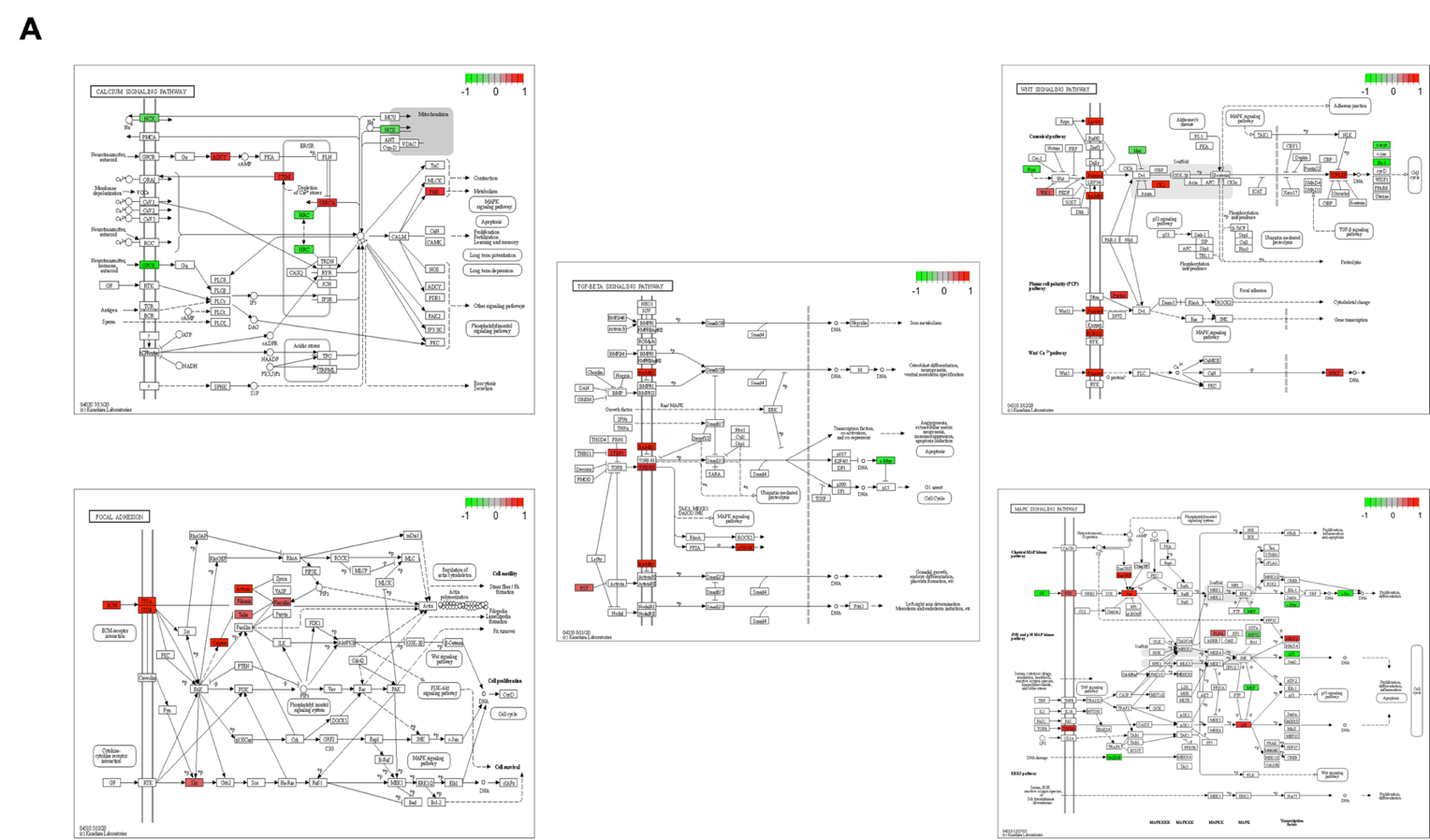


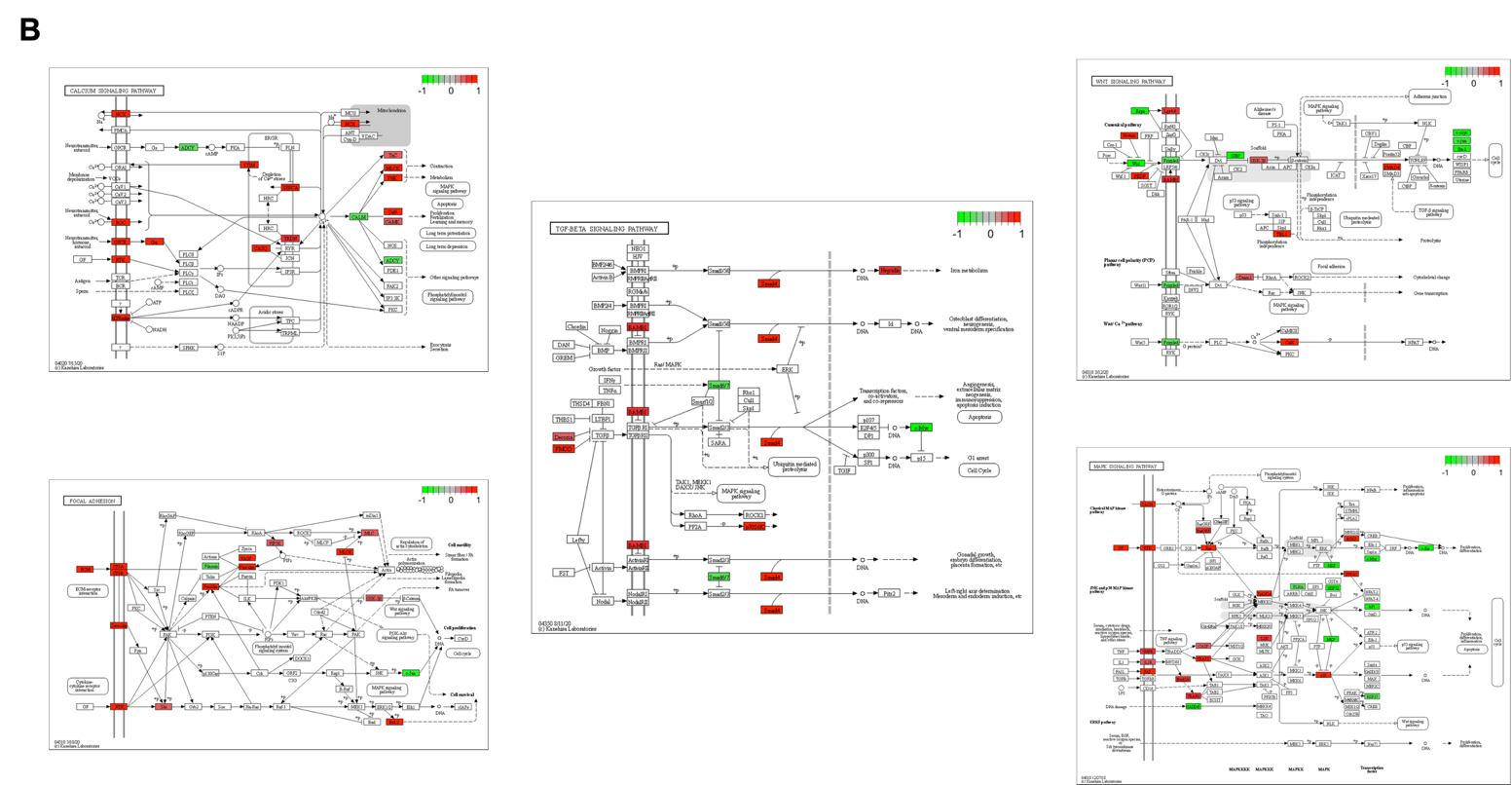


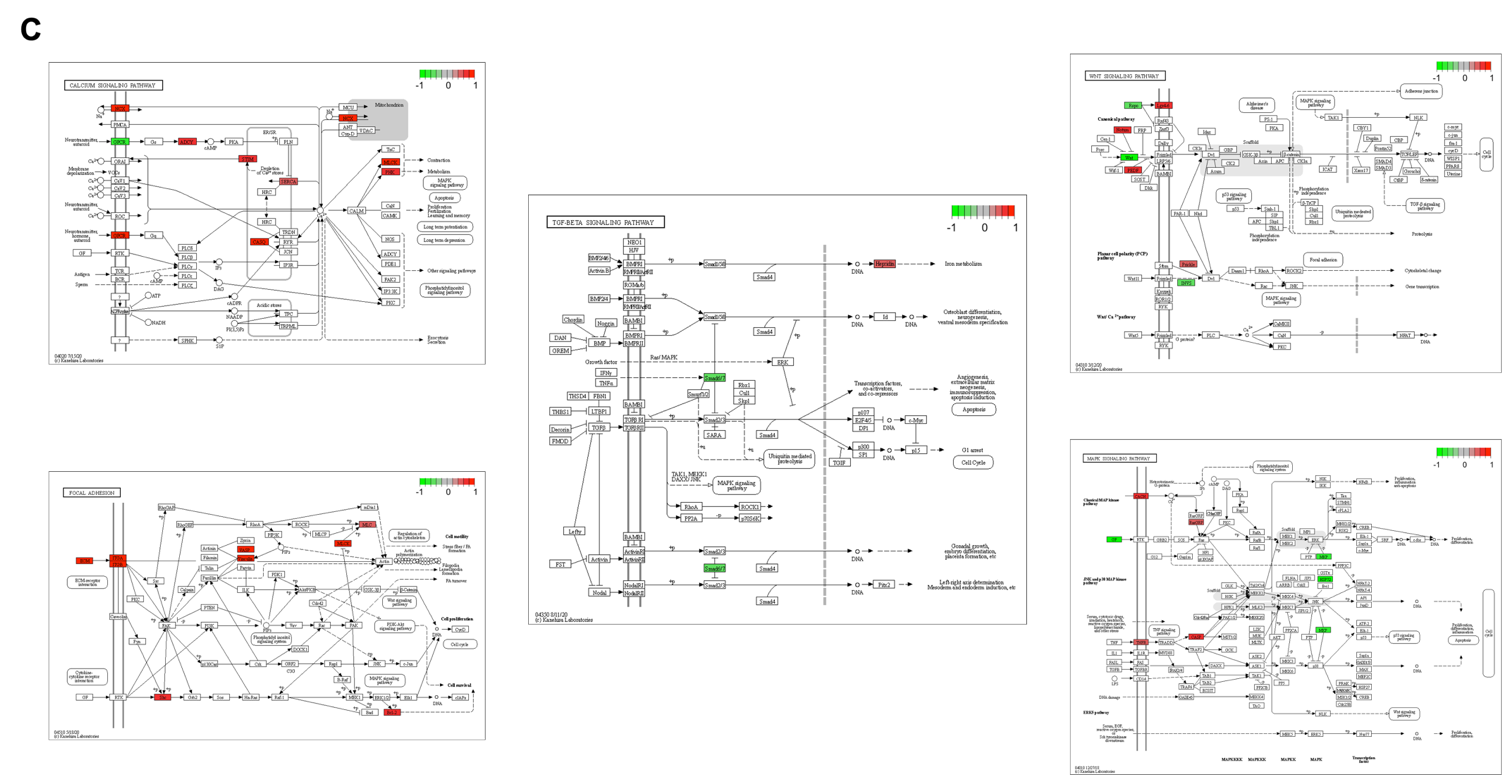


**Supplementary Figure 3- Validation of RNA-Seq based expression patterns by RT-PCR using eight selected genes from MAPK pathway involved in skeletal development. Bar plots indicate the log2 fold change values for different genes across different treatments compared to control. Ribosomal protein L13a (*rpl13a*) and ribosomal protein, large, P0 (*rplp0*) were used as the housekeeping genes in RT-PCRs to standardize the results by eliminating variation in mRNA and cDNA quantity or quality.**
